# An SETD1A/Wnt/β-catenin feedback loop promotes NSCLC development

**DOI:** 10.1186/s13046-021-02119-x

**Published:** 2021-10-13

**Authors:** Rui Wang, Jian Liu, Kai Li, Ganghua Yang, Sisi Chen, Jie Wu, Xinming Xie, Hong Ren, Yamei Pang

**Affiliations:** 1 Department of Thoracic Surgery, the First Affiliated Hospital of Xi’an Jiaotong University, 277 Yanta West Road, Xi’an, Shaanxi 710061 P.R. China; 2 Department of Respiratory and Critical Care Medicine, the First Affiliated Hospital of Xi’an Jiaotong University, Xi’an, 277 Yanta West Road, Xi’an, 710061 Shaanxi Province China; 3 Department of Geriatric Surgery, the First Affiliated Hospital of Xi’an Jiaotong University, 277 Yanta West Road, Xi’an, Shaanxi 710061 People’s Republic of China

**Keywords:** SETD1A, β-Catenin, NEAT1, EZH2, NSCLC

## Abstract

**Background:**

SETD1A, a member of SET1/MLL family H3K4 methyltransferases, is involved in the tumorigenesis of numerous cancers. However, the biological role and mechanism of SETD1A in non-small cell lung cancer (NSCLC) remain to be elucidated.

**Methods:**

The expression of SETD1A, NEAT1, EZH2, and β-catenin in NSCLC tissues and cell lines was detected by qRT-PCR, immunohistochemistry and western blotting. The regulatory mechanisms were validated by chromatin immunoprecipitation, co-immunoprepitation and luciferase reporter assay. The self-renewal, cisplatin sensitivity and tumorigenesis of NSCLC cells were analyzed using sphere formation, CCK-8, colony formation assays and xenograft tumor models.

**Results:**

SETD1A expression was significantly increased in NSCLC and its overexpression predicted a poor prognosis of patients with NSCLC. Functional experiments showed that SETD1A positively regulated cancer stem cell property and negatively regulated cisplatin sensitivity in NSCLC cells via the Wnt/β-catenin pathway. Next, we found that SETD1A positively regulated the Wnt/β-catenin pathway via interacting with and stabilizing β-catenin. The SET domain is dispensable for the interaction between SETD1A and β-catenin. Furthermore, we identified that SETD1A bound to the promoters of NEAT1 and EZH2 to activate gene transcription by inducing H3K4me3 enrichment. Rescue experiments showed that SETD1A promoted the Wnt/β-catenin pathway and exerted its oncogenic functions in NSCLC, at least, partly through NEAT1 and EZH2 upregulation. In addition, SETD1A was proven to be a direct target of the Wnt/β-catenin pathway, thus forming a positive feedback loop in NSCLC cells.

**Conclusion:**

SETD1A and Wnt/β-catenin pathway form a positive feedback loop and coordinately contribute to NSCLC progression.

**Supplementary Information:**

The online version contains supplementary material available at 10.1186/s13046-021-02119-x.

## Background

Lung cancer, one of the most prevalent malignant tumors, is classified into two categories according to pathological characteristics, namely small cell lung cancer and non-small cell lung cancer (NSCLC) [1]. Substantial advances in the treatment of NSCLC have been achieved in the past few years due to the widely use of molecular targeted therapy and immunotherapy; however, the five-year survival rate of lung cancer is still less than 20% [2, 3]. Thus, further investigations on the underlying mechanisms of NSCLC genesis and progression remain urgent clinical requirement.

Histone 3 Lysine 4(H3K4) methylation, including monomethylation, bimethylation and trimethylation (H3K4me1/2/3), is generally considered to be a transcriptional activation mark due to its capability to recruit transcription factors/coactivators [4]. The methylation of H3K4 in mammalian cells is catalyzed by SET1/MLL family H3K4 methyltransferases, including SETD1A, SETD1B, MLL1, MLL2, MLL3, MLL4 and MLL5 [5]. SETD1A prefers to catalyze H3K4 to H3K4me3, which is commonly enriched at transcription start sites to activate gene transcription [4, 6]. SETD1A is involved in pluripotency and malignant transformation of stem cells [7–10]. Additionally, SETD1A is overexpressed and promotes malignant phenotypes in many cancers, such as breast cancer [11, 12], acute myeloid leukemia [13], colorectal cancer [6], gastric cancer [14], hepatocellular cell carcinoma [15] and prostate cancer [16]. Furthermore, SETD1A methylates YAP at K342 to increase YAP activity, leading to increased cell proliferation and tumorigenesis in hepatocellular cell carcinoma and lung cancer [15, 17]. However, the biological role and mechanism of SETD1A in NSCLC remains largely unclear.

Aberrant activation of the Wnt/β-catenin pathway is a critical event in the genesis of various cancers [18]. Β-catenin, the pivotal component of the Wnt pathway, is tightly regulated at many hierarchical levels, including CTNNB1 transcription, protein stability, subcellular localization and transcriptional activity of β-catenin [19]. Li et al. have reported that HIRA enhances CTNNB1 transcription by recruiting SETD1A, which increases H3K4me3 levels in the CTNNB1 promoter, thereby activating the Wnt/β-catenin pathway in neural progenitor cells [20]. Two other studies have demonstrated that SETD1A cooperates with β-catenin to activate the transcription of Wnt/β-catenin target genes in an H3K4me3-dependent manner in embryonic stem cells and cancer cells [6, 21]. Although SETD1A has been proven to activate the Wnt/β-catenin pathway, the interplay between SETD1A and the Wnt/β-catenin pathway in cancer cells remains to be elucidated.

In this study, we found that SETD1A regulates cancer stem cell property and cisplatin sensitivity in NSCLC cells via activating the Wnt/β-catenin pathway. Mechanistically, SETD1A activates the Wnt/β-catenin pathway via interacting with and stabilizing β-catenin. Furthermore, we identified two direct SETD1A target genes, NEAT1 and EZH2, through which SETD1A promotes Wnt/β-catenin pathway activity and malignant behaviors. Importantly, we demonstrated that SETD1A is a direct target of the Wnt/β-catenin pathway, thus forming a feedback loop to promote NSCLC progression.

## Methods

### Cell culture and reagent

Lung cancer cell lines (A549 and PC9), HEK293T cell line and normal bronchial epithelial cell line (BEAS2B) were purchased from cell banks of Shanghai Institutes of Biological Sciences (Shanghai, China). A549, HEK293T and BEAS2B cell lines were cultured in DMEM (HyClone, Logan, Utah, USA) with 10% fetal bovine serum (FBS; Gibco, Carlsbad, CA, USA) and antibiotics (100 U/mL penicillin and 100 μg/mL streptomycin). PC9 cells were cultured in RPMI 1640 medium (HyClone) with 10% FBS and antibiotics. All the cells were maintained in a humidified atmosphere of 5% CO_2_ at 37 °C. Recombinant Wnt3a was purchased from R&D Systems (Minneapolis, MN, USA).

### Plasmid construction

The full-length sequences of SETD1A, β-catenin, NEAT1 and EZH2 were inserted into the pcDNA3.1 vector (Invitrogen, Carlsbad, CA, USA) to construct the SETD1A, β-catenin, NEAT1 and EZH2-overexpressing plasmids, respectively. The NEAT1, EZH2 and SETD1A promoter fragments (2 kb, 2 kb and 0.4 kb) were inserted into the pGL3-basic vector (Promega, Madison, WI, USA) to construct luciferase activity reporter plasmids (NEAT1-P-wt, EZH2-P-wt and SETD1A-P-wt) for the promoter activity assay, respectively. The TCF/LEF binding site mutated SETD1A promoter reporter plasmid (SETD1A-P-mut) and mutant β-catenin plasmid with K312E and K435E mutations (β-catenin-DM) were generated by site-specific mutagenesis based on the SETD1A-P-wt plasmid and wild type β-catenin plasmid, respectively. The TOP flash plasmid, FOP flash plasmid and pRL-TK vector were obtained from MiaolingBio (Wuhan, Hubei, China). A series of plasmids of Flag-tagged SETD1A mutants were generated by YouBio (Wuhan, Hubei, China).

### Cell transfection

Β-catenin small interfering RNA (siRNA) and the corresponding negative control were synthesized by GenePharma (Shanghai, China). The transfection of plasmids and siRNA was performed using Lipo8000™ transfection reagent (C0533; Beyotime, Shanghai, China) according to the manufacturer’s instruction. The lentiviruses delivering short hairpin RNAs (shRNAs) targeting SETD1A and scrambled control sequences were obtained from GeneChem (Shanghai, China). The target sequences of shRNA and siRNA are listed in Additional file 1: Table S1.

### Tissue specimens

Clinical samples were obtained from the First Affiliated Hospital of Xi’an Jiaotong University. This study was approved by the Ethics Committee of the First Affiliated Hospital of Xi’an Jiaotong University. Each participant signed a written informed consent form. The clinical and pathological information of the patients are provided in Additional file 2: Table S2.

### Analysis of the online database

The RNA sequencing data of lung adenocarcinoma (containing 535 cancer tissues and 59 normal lung tissues) and lung squamous cell carcinoma (containing 502 cancer tissues and 49 normal lung tissues) were downloaded from The Cancer Genome Atlas (TCGA) database (https://tcga-data.nci.nih.gov/tcga/). The GSE71498 [22] and GSE52230 [6] datasets were downloaded from the Gene Expression Omnibus (GEO) database.

### Quantitative Realtime PCR (QRT-PCR)

Total RNA was isolated using a Total RNA extreme speed extraction kit (Fastagen, Shanghai, China) according to the manufacturer’s instructions. The extracted RNA was reverse transcribed using EasyQuick RT MasterMix (CWbio, Beijing, China). Realtime PCR was performed using 2 × RealStar Green Fast Mixture (Genstar, Beijing, China) on a CFX96 RealTime PCR system (Bio-Rad, Hercules, CA, USA) according to the manufacturer’s instructions. The relative expression levels were calculated using the 2^−ΔΔCt^ method as previously described [23]. The primer sequences are listed in Additional file 1: Table S1.

### Western blotting

Total protein was extracted using SDS lysis buffer (Beyotime). The nuclear and cytoplasmic protein was isolated using Nuclear Protein Extraction Kit (Solarbio, Beijing, China) according to the manufacturer’s instruction. The protein was separated by SDS-PAGE and transferred to polyvinylidene fluoride membranes (Millipore, Billerica, MA, USA). The membrane was blocked with 5% non-fat milk and incubated with primary antibodies at 4 °C overnight. Next, the membrane was washed 3 times and incubated with secondary antibodies for 90 min at 37 °C. The protein bands were visualized using an enhanced chemiluminescence kit (Millipore) on a ChemiDoc XRS+ imaging system (Amersham Biosciences, Uppsala, Sweden). The primary antibodies used for western blotting were as follows: anti-SETD1A (sc-515,590; Santa Cruz Biotechnology, Santa Cruz, CA, USA), anti-EZH2 (#5246; Cell Signaling Technology, Danvers, MA, USA), anti-CD133 (18470–1-AP; Proteintech, Wuhan, Hubei, China), anti-OCT4 (ab181557; Abcam, Cambriambridge, MA, USA), anti-SOX2 (11064–1-AP; Proteintech), anti-β-catenin (51067–2-AP; Proteintech), anti-CCND1 (sc-8396; Santa Cruz Biotechnology), anti-MYC (10828–1-AP; Proteintech), anti-phospho-β-Catenin (Ser675) (#4176; Cell Signaling Technology), anti-phospho-β-Catenin (Ser33/37/Thr41) (#9561; Cell Signaling Technology), anti-Ubiquitin (sc-8017; Santa Cruz Biotechnology), anti-PKAα cat (A-2) (sc-28,315; Santa Cruz Biotechnology), anti-APC (F-3) (sc-9998; Santa Cruz Biotechnology), mouse anti-Flag (F1804; Sigma-Aldrich), rabbit anti-Flag (AF0036; Beyotime), anti-HA (51064–2-AP; Proteintech), anti-GSK3α/β (sc-7291; Santa Cruz Biotechnology), anti-ICAT (ab129001; Abcam), anti-AXIN2 (20540–1-AP; Proteintech), anti-DKK1 (ab109416; Abcam), anti-GAPDH (10494–1-AP; Proteintech), anti-Vinculin (sc-73,614; Santa Cruz Biotechnology) and anti-Lamin B1 (sc-374,015; Santa Cruz Biotechnology).

### Immunofluorescence

NSCLC cells were fixed with 95% ethyl alcohol at room temperature for 30 min and permeabilized with 0.2% Triton X-100 (Beyotime) for 15 min. After blocking with 5% BSA (Solarbio), the cells were incubated with the anti-Setd1A antibody (sc-515,590; Santa Cruz Biotechnology), anti-β-catenin antibody (sc-7963; Santa Cruz Biotechnology) normal rabbit IgG (#2729; Cell Signaling Technology) and normal mouse IgG (A7028; Beyotime) at 4 °C overnight. Next, the cells were incubated with Alexa Fluor 488-labeled Goat Anti-Rabbit IgG (H + L) (A0423; Beyotime) and Cy3-labeled Goat Anti-Mouse IgG (H + L) (A0521; Beyotime) for 1 h at room temperature. After that, the cells were stained with DAPI (C1002; Beyotime) to visualize the nuclei. Finally, the cells were viewed and imaged under a laser scanning confocal microscope (Leica, Bensheim, Germany).

### Immunohistochemical (IHC) staining assay

Immunohistochemical staining was conducted as previously described [24]. The antibodies used for immunochemistry staining were as follows: anti-SETD1A (A300-289A; Bethyl Lab, Montgomery, TX, USA), anti-EZH2 (#5246; Cell Signaling Technology), anti-β-catenin (51067–2-AP; Proteintech), anti-Ki67 (27309–1-AP; Proteintech) and normal rabbit IgG (#2729; Cell Signaling Technology). Both the staining intensity and proportion of positive cells were taken into consideration: 0–25%, 26–50%, 51–75%, and 76–100% SETD1A positive cells were scored as 1, 2, 3, and 4, respectively; non-significant staining, light staining, moderate staining and strong staining were scored as 1, 2, 3, and 4, respectively. Then the two scores were multiplied to acquire a final score ranged from 1 to 16.

### Cisplatin sensitivity assay

The In vitro cisplatin sensitivity of NSCLC cells was determined by Cell Counting Kit-8 (CCK-8; Beyotime) according to the manufacturer’s protocol. NSCLC cells (5 × 10^3^) were seeded in 96-well plates overnight and subsequently incubated with the indicated cisplatin concentrations for 48 h. The final concentrations of cisplatin were 0 μM, 1 μM, 2 μM, 4 μM, 8 μM, 16 μM and 32 μM, respectively. Next, 10 μL of CCK-8 solution was added to each well and incubated for 2 h at 37 °C. The absorbance at a wavelength of 450 nm was measured using a microplate reader (Bio-Rad).

### Colony formation assay

NSCLC cells were seeded into 6-well dishes at a density of 1000 cells/well. For the next 14 days, culture media containing 5 μM cisplatin was replaced every 3 ~ 4 days until visible colonies had developed. The colonies were washed with 1 × PBS, fixed with cold 95% methanol and stained with 0.5% crystal violet. Colonies containing more than 50 cells were counted under a light microscope.

### Sphere formation assay

NSCLC cells were seeded in the 6-well ultra-low attachment plate (Corning, Lowell, MA, USA) at a density of 1000 cells/well. The cells were cultured in DMEM/F-12 medium (HyClone) supplemented with 20 ng/mL epidermal growth factor (EGF; Sigma-Aldrich, St. Louis, MO, USA), 10 ng/mL basic fibroblast growth factor (FGF; Sigma-Aldrich), 5 μg/mL insulin (Invitrogen) and B-27™ Supplement (Invitrogen) for 14 days. Then the spheres larger than 50 μm in diameter were counted and photographed under a light microscope [25, 26].

### Chromatin immunoprecipitation (ChIP) assay

ChIP assay was performed using EpiQuik™ Chromatin Immunoprecipitation Kit (P-2002; Epigentek, Farmingdale, NY, USA) according to the manufacturer’s instructions. NSCLC cells were cross-linked and sonicated as previously described [24]. The sheared chromatin fragments (approximately 200–500 bp) were subjected to immunoprecipitation with ChIP-grade antibodies as follows: anti-SET1AD antibody (A300-289A; Bethyl Lab), anti-H3K4me3 antibody (ab8580; Abcam), anti-β-catenin (51067–2-AP; Proteintech), anti-WDR5 antibody (#13105; Cell Signaling Technology), anti-H3K27me3 antibody (39,157; Active Motif), anti-H3K27ac antibody (39,134; Active Motif) and normal Rabbit IgG (#2729; Cell Signaling Technology). The primers for ChIP-PCR were listed in Additional file 1: Table S1.

### Co-immunoprecipitation (CoIP) assay

The CoIP assay was performed using Protein A/G PLUS-Agarose (sc-2003; Santa Cruz) according to the manufacturer’s instructions. For two-step CoIP, the first IP was performed using anti-Flag M2 beads (Sigma-Aldrich). Then the complex was eluted with 3 × Flag peptide (250 μg/ml, Sigma-Aldrich) and subjected to the second IP with anti-HA or control IgG. The protein samples from each step were analyzed by western blot analysis. The antibodies used for CoIP assay were anti-Setd1A antiboty (A300-289A; Bethyl Lab) and anti-β-catenin antibody (51067–2-AP; Proteintech), mouse anti-Flag (F1804; Sigma-Aldrich), anti-HA (51064–2-AP; Proteintech) and normal rabbit IgG (#2729; Cell Signaling Technology).

### Luciferase activity assay

A549 cells (1 × 10^5^) were plated in 24-well plates overnight and co-transfected with the indicated plasmid/siRNA or corresponding negative control and wild-type or mutant luciferase reporter vectors using Lipo8000™ Transfection Reagent. After transfection for 48 h, firefly and *Renilla* luciferase activities were measured using the Dual Luciferase Reporter Gene Assay Kit (Beyotime).

### In vivo xenograft assay

Four-week-old male BALB/cA-nu/nu mice were purchased from Beijing Huafukang Biosciences (Beijing, China). The animal experiments were approved by the Experimental Animal Committee of Xian Jiaotong University. For tumor propagation assay, PC-9 cells (2 × 10^6^) stably transfected with shSETD1A or scramble lentivirus were suspended in 100 μL of serum-free RPMI-1640 medium and subcutaneously inoculated into the axillary of mice (*n* = 5). The tumor volumes were measured every 3 days and calculated with the formula: V = 1/2 × L × W^2^ (L: long axis, W: short axis). All the mice were sacrificed after 28 days. The tumors were resected and subjected to immunohistochemical staining and qRT-PCR analysis. For tumor initiating assay, 1 × 10^5^, 1 × 10^4^ or 1 × 10^3^ SETD1A knockdown or negative control cells were subcutaneously injected into 4-week-old mice (*n* = 6). Tumor formation was monitored weekly for 8 weeks. The tumor initiating cells ratio or cancer stem cells frequency was calculated using the extreme limiting dilution analysis (http://bioinf.wehi.edu.au/software/elda/).

### Statistical analysis

All the experiments were performed in triplicate at least. All data were analyzed using GraphPad Prism 8 software (GraphPad Software, San Diego, CA, USA). Statistical *P* values were analyzed by a two-tailed Student’s *t*-test unless otherwise specified. Kaplan-Meier survival analysis was used to compare lung cancer patient survival by the log rank test. The results were considered statistically significant when *P* < 0.05.

## Results

### High expression of SETD1A correlates with poor prognosis in NSCLC patients

**Fig. 1:**
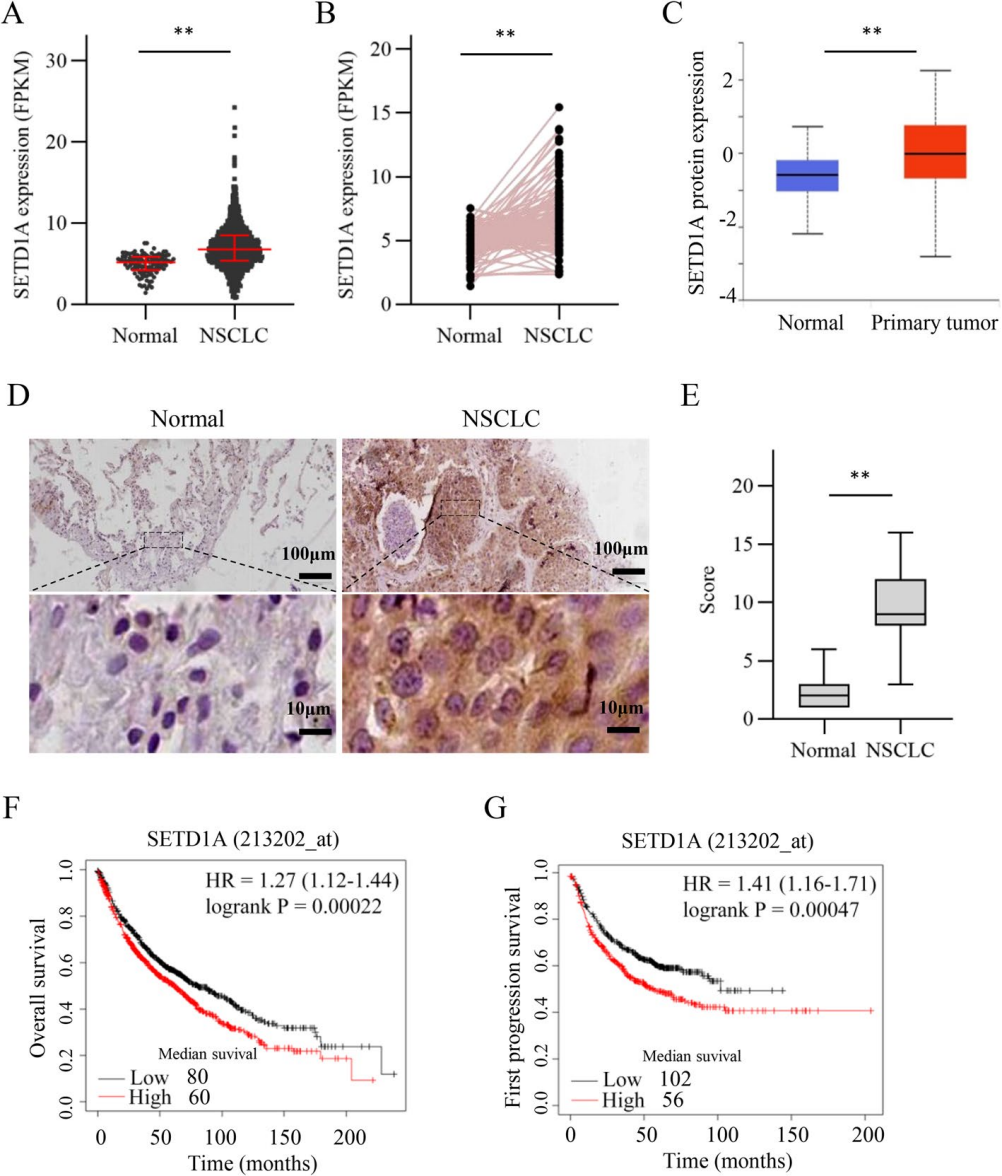
High expression of SETD1A correlates with a poor prognosis in NSCLC patients. **A**, SETD1A transcript levels in normal lung tissues (*n* = 108) and NSCLC tissues (*n* = 1037) from TCGA. NSCLC group consists of 535 LUAD tissues and 502 LUSC tissues. Data are presented as median (interquartile). Significance was determined using Mann-Whitney U test. **B**, SETD1A transcript levels in paired normal lung tissues (*n* = 106) and NSCLC tissues (n = 106) from TCGA. **C**, SETD1A protein expression in normal lung tissues (*n* = 111) and primary NSCLC tissues (n = 111) from CPTAC. **D**, Representative images of immunohistochemical (IHC) staining of SETD1A in normal lung tissues and NSCLC tissues. Scale bar, upper 100 μm, lower 10 μm. **E**, Quantification of SETD1A protein expression according to IHC scores in normal lung tissues and NSCLC tissues. **F**-**G**, Patients with high SETD1A expression have a poorer overall survival (**F**) and first progression survival (**G**) according to Kaplan Meier-plotter (http://kmplot.com/analysis/index.php?p=service&cancer=lung). **P < 0.01.

To investigate the role of SETD1A in NSCLC, we first employed RNA sequencing data from TCGA database to validate the expression SETD1A in NSCLC. We found that SETD1A levels were increased in NSCLC tissues compared with those in the normal lung tissues (Fig. 1A). The results were further confirmed in paired NSCLC and normal lung tissues from TCGA database (Fig. 1B). The CPTAC (clinical proteomic tumor analysis consortium) analysis showed that SETD1A protein levels were higher in primary NSCLC tissues than in normal lung tissues on the ULCAN website (Fig. 1C). To further validate the expression of SETD1A protein in NSCLC, we conducted immunochemistry staining of clinical specimens. Immunohistochemical staining analysis showed that SETD1A protein was highly expressed in NSCLC tissues compared with that in normal lung tissues (Fig. 1D-E and Additional file 3: Fig. S1). Moreover, high SETD1A expression was significantly associated with a poor overall survival and first progression survival (Fig. 1F-G). These results suggest that SETD1A is associated with NSCLC progression.

### SETD1A regulates cancer stem cell property and cisplatin sensitivity in NSCLC cells

**Fig. 2:**
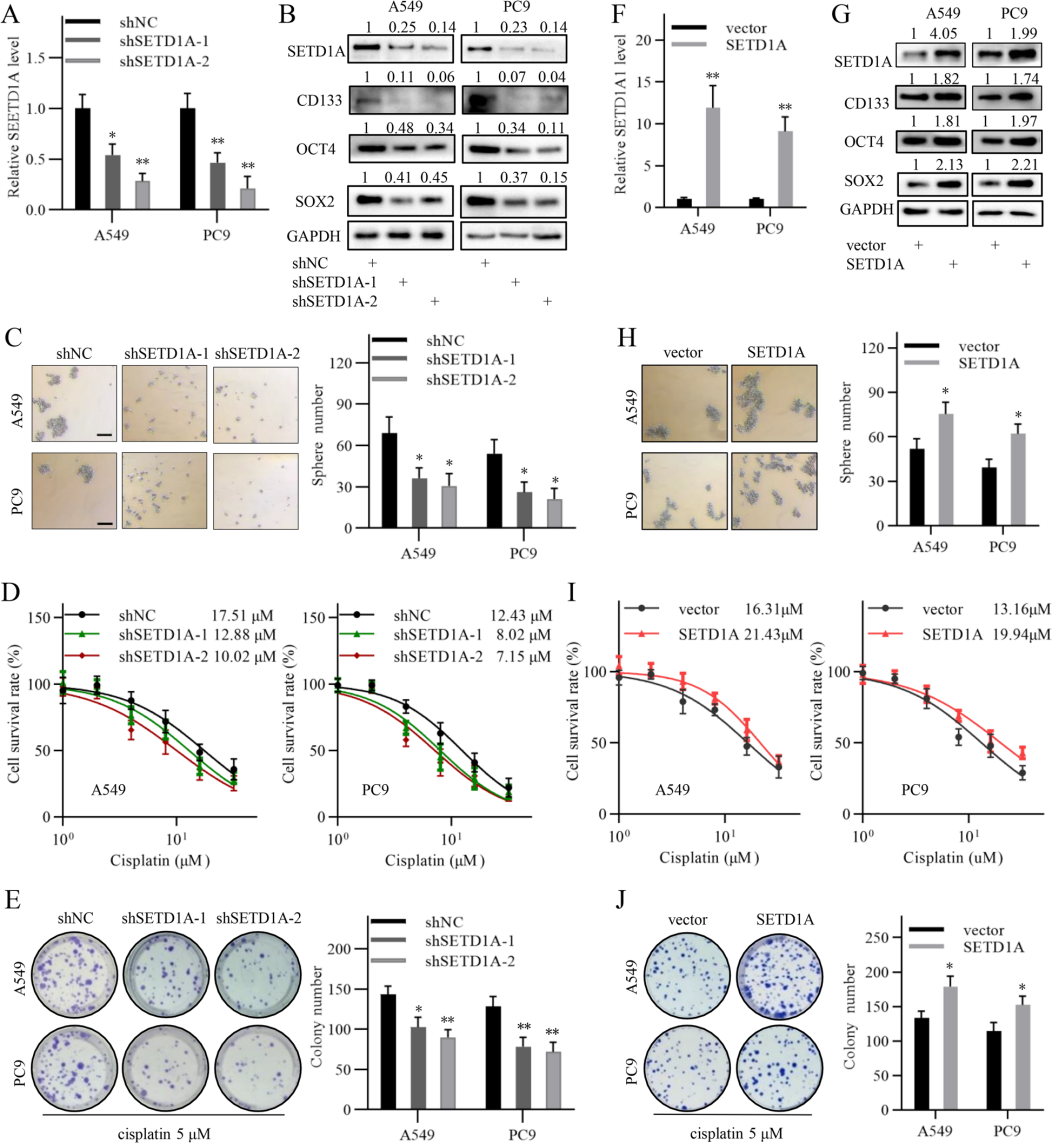
SETD1A regulates cancer stem cell property and cisplatin sensitivity in NSCLC cells. **A**, QRT-PCR analysis of SETD1A transcript expression in NSCLC cells transfected with shNC or shSETD1A lentivirus. **B**, SETD1A, SOX2, OCT4 and CD133 protein expression in NSCLC cells in the SETD1A knockdown and negative control groups was analyzed by western blotting. **C**, Sphere formation assay of the SETD1A knockdown and negative control NSCLC cells. Scale bar, 100 μm. **D**, Cisplatin sensitivity of the SETD1A knockdown and negative control NSCLC cells was analyzed by CCK-8 assay. The final concentrations of cisplatin were 1 μM, 2 μM, 4 μM, 8 μM, 16 μM and 32 μM. **E**, The growth of the SETD1A knockdown and negative control NSCLC cells exposed to cisplatin treatment was analyzed by colony formation assay. The final concentration of cisplatin was 5 μM. **F**, QRT-PCR analysis of SETD1A transcript expression in NSCLC cells transfected with the empty vector or SETD1A expression vector. **G**, SETD1A, SOX2, OCT4 and CD133 protein expression in the NSCLC cells transfected with empty vector or SETD1A expression vector was analyzed by western blotting. **H**, Sphere formation assay of NSCLC cells transfected with the empty vector or SETD1A expression vector. Scale bar, 100 μm. **I**, Cisplatin sensitivity of the NSCLC cells transfected with the empty vector or SETD1A expression vector was analyzed by CCK-8 assay. **J**, The growth of the NSCLC cells transfected with empty vector or SETD1A expression vector was analyzed by colony formation assay. The final concentration of cisplatin was 5 μM. Data are shown as means ± SD. *P < 0.05, **P < 0.01.

To investigate the function of SETD1A in NSCLC cells, we used two shRNAs to stably knock down SETD1A expression in NSCLC cell lines (Fig. 2A-B). Western blot analysis showed that the expression of cancer stem cell related genes, such as SOX2, OCT4 and CD133, was reduced following SETD1A knockdown (Fig. 2B). Sphere formation assays showed that SETD1A knockdown significantly reduced the self-renewal ability of NSCLC stem cells (Fig. 2C). Furthermore, CCK-8 assays showed that SETD1A knockdown significantly increased the sensitivity of NSCLC cells to cisplatin treatment (Fig. 2D and Additional file 4: Fig. S2A). Colony formation assays showed that SETD1A knockdown significantly reduced the growth of NSCLC cells exposed to cisplatin treatment (Fig. 2E). In contrast, functional experiments showed that SETD1A overexpression exhibited opposite effects on cancer stem cell property and cisplatin sensitivity in NSCLC cells (Fig. 2F-J and Additional file 4: Fig. S2B). These results suggest that SETD1A regulates cancer stem cell property and cisplatin sensitivity in NSCLC cells.

### SETD1A promotes Wnt/β-catenin pathway activity via increasing β-catenin stability

**Fig. 3:**
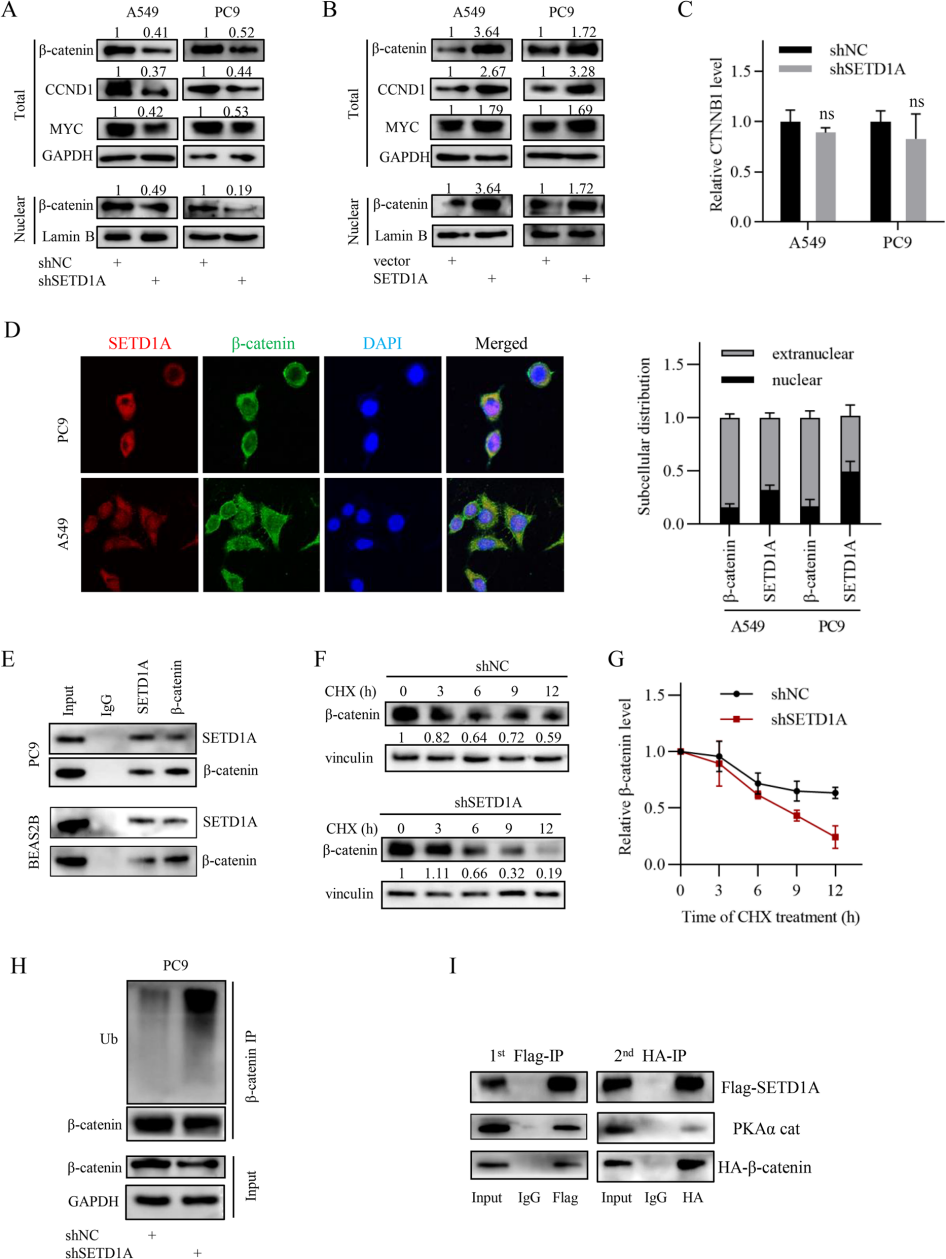
SETD1A activates the Wnt/β-catenin pathway by stabilizing β-catenin. **A-B**, MYC, CCND1 and nuclear β-catenin levels in NSCLC cells as indicated were analyzed by western blotting. **C**, CTNNB1 transcript levels in NSCLC cells as indicated was analyzed by qRT-PCR. ns, not significant. **D**, Subcellular localization of SETD1A and β-catenin in NSCLC cells was analyzed by confocal laser scanning microscope. The subcellular distribution of SETD1A and β-catenin was quantified by Image J software. **E**, The interaction between SETD1A and β-catenin in PC9 cells was analyzed by CoIP assay. **F**-**G**, β-catenin stability in the SETD1A knockdown and negative control group PC9 cells was analyzed by CHX chase assay. **H**, Ubiquitination of β-catenin in PC9 cells was analyzed by CoIP assay following SETD1A knockdown. **I**, Two-step co-immunoprecipitation of the complex containing SETD1A, β-catenin and PKA is shown. HEK293T cells were cotransfected with the Flag-tagged SETD1A plasmid and HA-tagged β-catenin plasmid. The first immunoprecipitation was performed using anti-FLAG M2 beads. The complex was eluted using 3 × Flag peptide followed by the second step of co-immunoprecipitation with anti-HA antibody. Then the protein samples were analyzed by western blotting with anti-Flag, anti-HA and anti- anti-PKAα cat antibody. Data are shown as means ± SD.

Previous studies have proven that SETD1A promotes Wnt/β-catenin pathway activity in colorectal cancer and neural progenitor cells [6, 20]. Thus, we further investigated whether SETD1A regulates cancer stem cell property and chemotherapy sensitivity in NSCLC via the Wnt/β-catenin pathway. We chose shSETD1A-2 for the following experiments due to its higher knockdown efficiency. Initially, we employed the TOP/FOP reporter system to assess Wnt/β-catenin pathway activity. The results showed that SETD1A positively regulated the Wnt/β-catenin pathway activity (Additional file 5: Fig. S3A). Western blot and immunofluorescence analysis showed that nuclear β-catenin was positively regulated by SETD1A (Fig. 3A-B and Additional file 5: Fig. S3B). Besides, it was further validated by the western blotting results of Wnt/β-catenin target genes (MYC and CCND1) expression (Fig. 3A-B). Subsequently, we demonstrated that β-catenin overexpression reversed the effects of SETD1A knockdown on cancer stem cell property and cisplatin sensitivity of NSCLC cells (Additional file 6: Fig. S4A-C). These results suggest that SETD1A promotes NSCLC progression via activating the Wnt/β-catenin pathway.

To explore the mechanism by which SETD1A regulates the Wnt/β-catenin pathway, we first examined the influence of SETD1A on β-catenin expression. We found that SETD1A positively regulated the total and cytoplasmic β-catenin protein levels, while the transcript levels were not significantly affected (Fig. 3A-C and Additional file 5: Fig. S3C-D). Furthermore, BUSCA webserver predicted that SETD1A protein might localize in both the cytoplasm and nucleus (Additional file 5: Fig. S3E). Consistently, immunohistochemical staining and immunofluorescence analysis showed that SETD1A localized in nucleus and cytoplasm (Figs. 1D, 3D and Additional file 5: Fig. S3F). Interestingly, confocal analysis showed that SETD1A protein colocalized with β-catenin not only in the nucleus but also in the cytoplasm, where the β-catenin protein is ubiquitinated and targeted for proteasome-dependent degradation (Fig. 3D and Additional file 5: Fig. S3F) [19]. Moreover, co-immunoprecipitation and reciprocal co-immunoprecipitaion analysis showed that endogenous SETD1A interacted with endogenous β-catenin in normal bronchial epithelial BEAS-2B cells and NSCLC cells (Fig. 3E). These results suggest that SETD1A may play a role in the protein turnover of β-catenin. To investigate whether SETD1A affects β-catenin protein stability, we conducted a cycloheximide (CHX) chase assay in SETD1A-knockdown and negative control cells. The results indicated that SETD1A positively regulated the protein stability of β-catenin (Fig. 3F-G and Additional file 5: Fig. S3G). The above results suggest that SETD1A interacts with and stabilizes β-catenin in NSCLC cells.

The phosphorylation and subsequent ubiquitination of β-catenin plays an important role in β-catenin stability [19]. Thus, we detected the β-catenin ubiquitination status following SETD1A knockdown. The results showed that SETD1A knockdown increased the ubiquitination of β-catenin (Fig. 3H). Consistently, MG132 treatment attenuated the inhibitory effect of SETD1A knockdown on the β-catenin expression (Additional file 5: Fig. S3H). Subsequently, we detected the phosphorylation status of β-catenin at some sites, such as S33/37/T41 and S675, which are involved in ubiquitination and degradation of β-catenin [27–29]. We found that S33/37/T41 phosphorylation was only slightly elevated following SETD1A knockdown in the presence of Wnt3a, accompanied by a slightly increased interaction between β-catenin and the destructive complex (AXIN2, GSK3β and APC) (Additional file 5: Fig. S3I). However, S675 phosphorylation was dramatically reduced following SETD1A knockdown, indicating that S675 phosphorylation might play a more predominant role in the regulation of β-catenin by SETD1A (Additional file 5: Fig. S3I). It was further validated by the increased S675 phosphorylation level induced by SETD1A overexpression (Additional file 5: Fig. S3J). PKA is reported to catalyze S675 phosphorylation of β-catenin [28]. Thus, we wondered whether PKA is involved in S675 phosphorylation induced by SETD1A. Immunoprecipitation analysis showed that SETD1A, β-catenin and PKA formed a trimeric complex, which might be responsible for β-catenin phosphorylation and stabilization induced by SETD1A (Fig. 3I).

### SETD1A binds to β-catenin via its SET domain

**Fig. 4:**
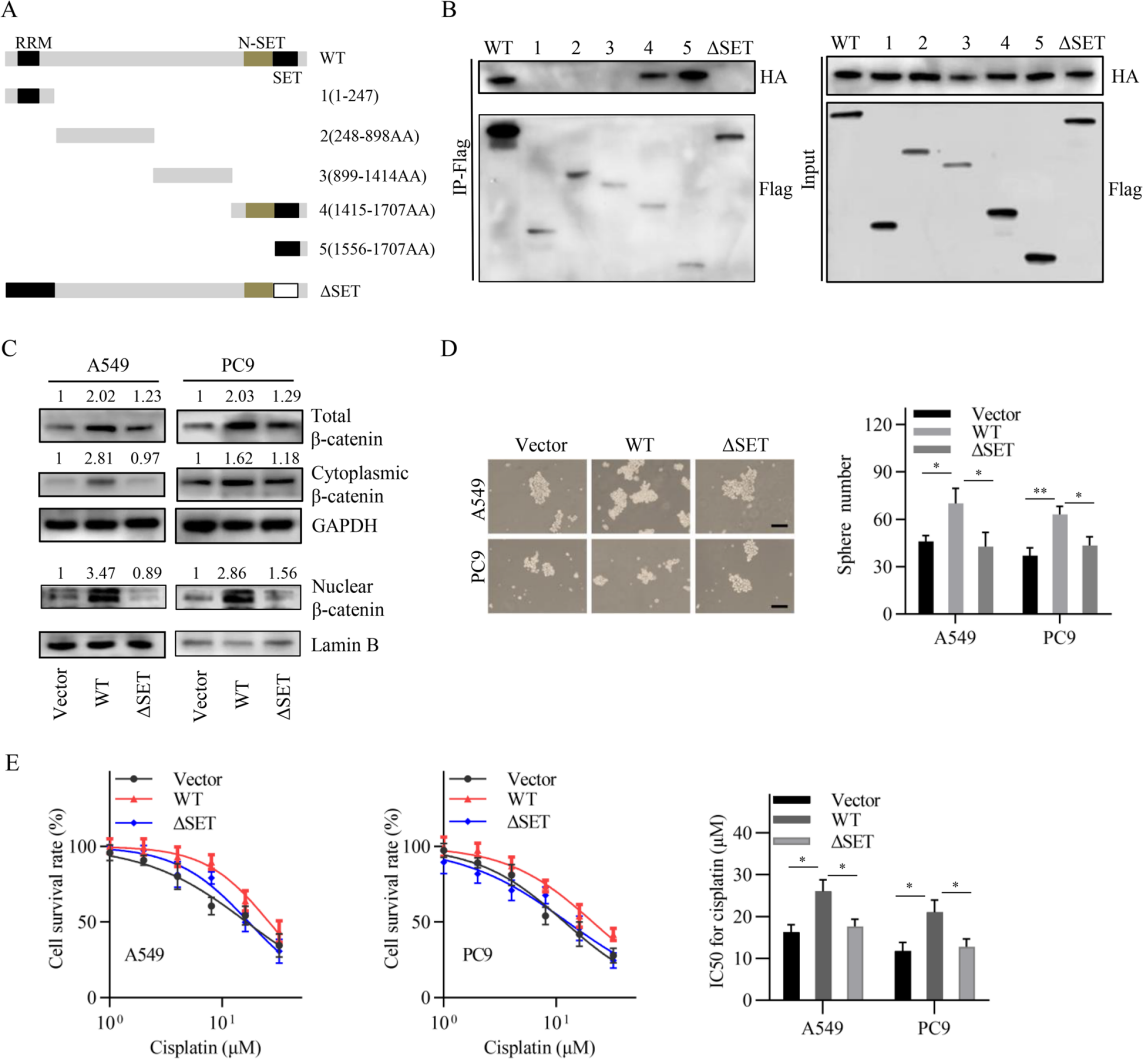
SETD1A binds to β-catenin via its SET domain, **A**, Schematic representation of SETD1A mutants. **B**, HEK293T cells were cotransfected with the indicated plasmids of Flag-tagged SETD1A mutants and HA-tagged full length β-catenin. Cell lysates were immunoprecipitated with anti-Flag antibody. **C**, The total, cytoplasmic and nuclear β-catenin levels were detected by western blot analysis following transfection with the empty vector, wild-type SETD1A plasmid and ΔSET plasmid. **D**, Sphere formation ability was determined following transfection with the empty vector, wild-type SETD1A plasmid and ΔSET plasmid. Scale bar, 100 μm. **E**, Cisplatin sensitivity was detected by the CCK-8 assay following transfection with the empty vector, wild type SETD1A plasmid and ΔSET plasmid. Data are shown as means ± SD. *P < 0.05, **P < 0.01.

We further investigated the domain of SETD1A responsible for binding to β-catenin. A series of plasmids of Flag-tagged SETD1A mutants and one HA-tagged β-catenin plasmid were used to perform immunoprecipitation assays in HEK293T cells (Fig. 4A). The results showed that SET domain was required for SETD1A binding to β-catenin (Fig. 4B). Deletion of the SET domain, at least, partly abolished the effects of SETD1A on the total, cytoplasmic and nuclear β-catenin levels, sphere formation and cisplatin sensitivity in NSCLC cells (Fig. 4C-E). These results suggest that the SET domain is dispensable for the interaction between SETD1A and β-catenin.

### SETD1A activates NEAT1 and EZH2 transcription to promote the Wnt/β-catenin pathway

**Fig. 5:**
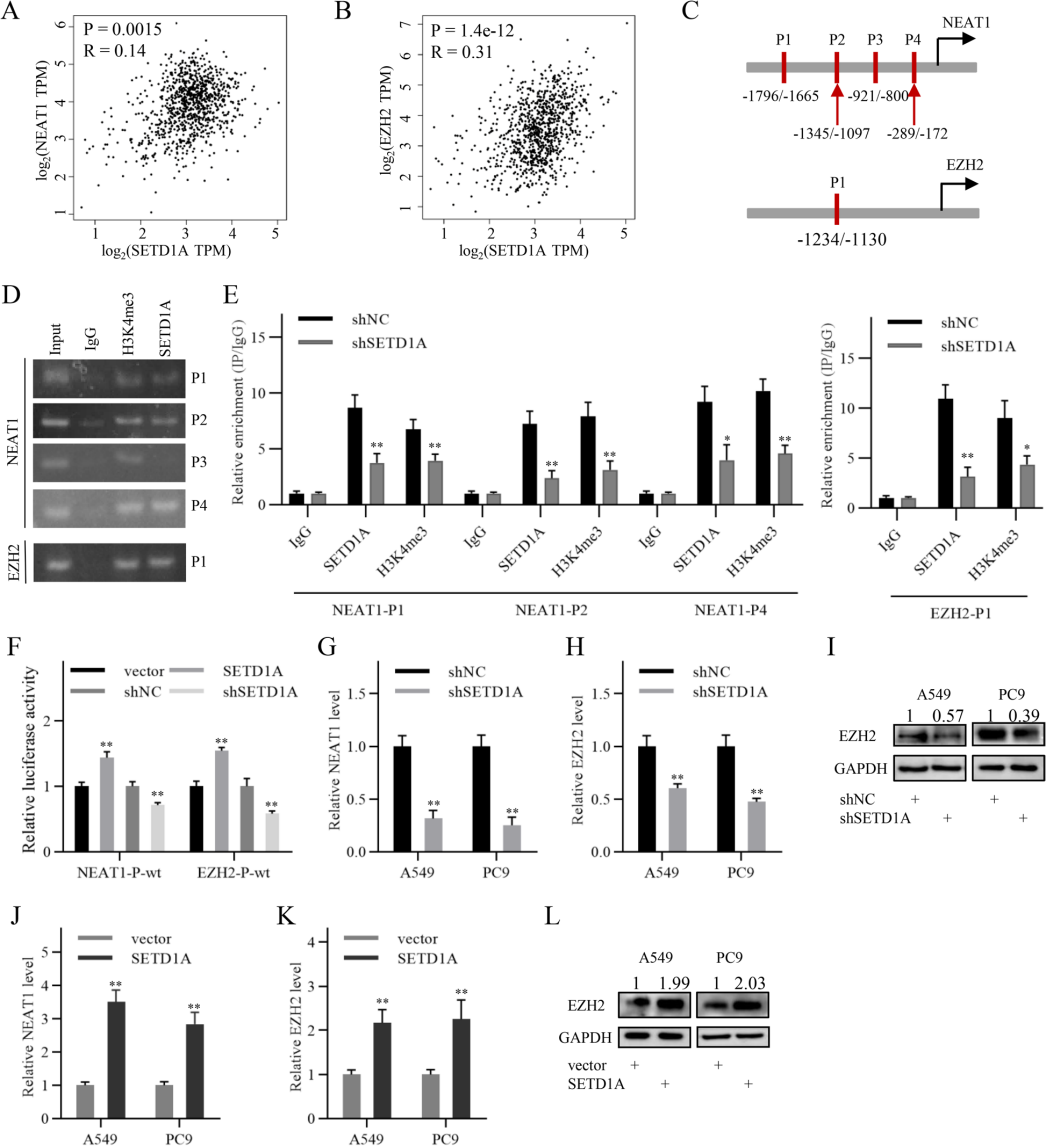
SETD1A activates NEAT1 and EZH2 transcription to activate the Wnt/β-catenin pathway. **A-B**, The correlation between SETD1A and NEAT1, EZH2 was analyzed using GEPIA online tool. R, Pearson’s correlation coefficient. **C**, Schematic showing the ChIP-PCR detection site in the NEAT1 and EZH2 promoters. **D**, The enrichment of SETD1A and H3K4me3 in the NEAT1 and EZH2 promoters in A549 cells was detected by ChIP-PCR assay. **E**, The relative enrichment of SETD1A and H3K4me3 in the NEAT1 and EZH2 promoters was detected by ChIP-qPCR assay following SETD1A knockdown in A549 cells. **F**, The promoter activity was analyzed by luciferase activity assay in A549 cells. **G**-**H**, NEAT1 and EZH2 transcript levels in NSCLC cells were detected by qRT-PCR following SETD1A knockdown. **I**, EZH2 protein levels in NSCLC cells as indicated were detected by western blotting following SETD1A knockdown. **J-K**, NEAT1 and EZH2 transcript levels in NSCLC cells were detected by qRT-PCR following SETD1A overexpression. **L**, EZH2 protein levels in NSCLC cells were detected by western blotting following SETD1A overexpression. Data are shown as means ± SD. *P < 0.05, **P < 0.01.

To further explore the mechanism by which SETD1A regulates the Wnt/β-catenin pathway, we analyzed the RNA sequencing data in the GSE71498 and GSE52230 datasets. We found that SETD1A knockdown in A549, MDA-MB-231 and HCT116 cells increased the expression of some Wnt/β-catenin negative regulators, such as AXIN2, ICAT, DKK1 and SIAH1 (Additional file 7: Fig. S5A-B). Next, we detected the effects of SETD1A knockdown on the expression of these negative regulators in NSCLC cells by qRT-PCR and western blotting. Another Wnt/β-catenin negative regulator, GSK3β, was also included in this study. The results showed that GSK3β and ICAT were increased following SETD1A knockdown in NSCLC cells (Additional file 7: Fig. S5C-E). Previous studies have reported that NEAT1 acts as a scaffold molecule of the polycomb protein complex containing EZH2 to repress the transcription of Wnt/β-catenin pathway negative regulators, including AXIN2, GSK3β and ICAT [30]. Furthermore, SETD1A expression in NSCLC tissues was positively correlated with NEAT1 and EZH2 expression according to GEPIA and StarBase online database analysis, respectively (Fig. 5A-B and Additional file 7: Fig. S5F-I). Therefore, we speculated that SETD1A might activate the Wnt/β-catenin pathway via increasing NEAT1 and EZH2 expression.

Subsequently, we investigated H3K4me3 enrichment in the NEAT1 and EZH2 promoters using ChIP sequencing data in ENCODE database. Visualization analysis by the UCSC genome browser showed that H3K4me3 peaks were enriched in the NEAT1 and EZH2 promoters, indicating that SETD1A might promote the transcription of NEAT1 and EZH2 in an H3K4me3-dependent manner (Additional files 8 and 8: Figs. S6 and S7). Next, we designed PCR primers corresponding to the H3K4me3 peak positions to perform ChIP-PCR assay (Fig. 5C). ChIP-PCR results showed that both SETD1A and H3K4me3 were associated with the NEAT1 and EZH2 promoters at the indicated sites (Fig. 5D). Furthermore, we chose the sites with enriched SETD1A and H3K4me3 to explore the effects of SETD1A knockdown on H3K4me3 enrichment in the promoters of NEAT1 and EZH2. ChIP-qPCR results showed that SETD1A knockdown significantly reduced the enrichment of H3K4me3 at the indicated sites (Fig. 5E, Additional file 10: Fig. S8). Next, we constructed two luciferase reporter plasmids with the promoter fragments of EZH2 and NEAT1, respectively. Luciferase activity assays showed that the activity of the NEAT1 and EZH2 promoters was positively regulated by SETD1A (Fig. 5F). Finally, the expression of the two genes was detected by qRT-PCR and western blotting. The transcript and protein levels of NEAT1 and EZH2 were reduced by SETD1A knockdown, while increased by SETD1A overexpression (Fig. 5G-L). These results suggest that SETD1A binds to the NEAT1 and EZH2 promoters to activate gene transcription in an H3K4me3-dependent manner.

To investigate whether NEAT1 and EZH2 are functional targets of SETD1A in NSCLC cells, we constructed a NEAT1 expression plasmid and an EZH2 expression plasmid. The transfection effects of the two plasmids were validated by qRT-PCR and western blotting (Additional file 11: Fig. S9A-B). Next, we transfected the two plasmids into the SETD1A-knockdown cell lines, respectively. Both EZH2 and NEAT1 overexpression attenuated the increased ICAT and GSK3β expression induced by SETD1A knockdown in NSCLC cells (Additional file 11: Fig. S9C). Additionally, the reduced Wnt/β-catenin pathway activity induced by SETD1A knockdown was restored (Additional file 11: Fig. S9D). Therefore, SETD1A promotes Wnt/β-catenin pathway activity via activating NEAT1 and EZH2 transcription. In addition, functional experiments showed that both NEAT1 and EZH2 overexpression attenuated the reduced self-renewal ability and increased cisplatin sensitivity induced by SETD1A knockdown (Additional file 6: Fig. S4A-B). These results suggested that SETD1A promotes the Wnt/β-catenin pathway and NSCLC progression, at least, partly via activating NEAT1 and EZH2 transcription.

### The SETD1A/NETA1/EZH2/β-catenin axis promotes NSCLC progression in vivo

**Fig. 6:**
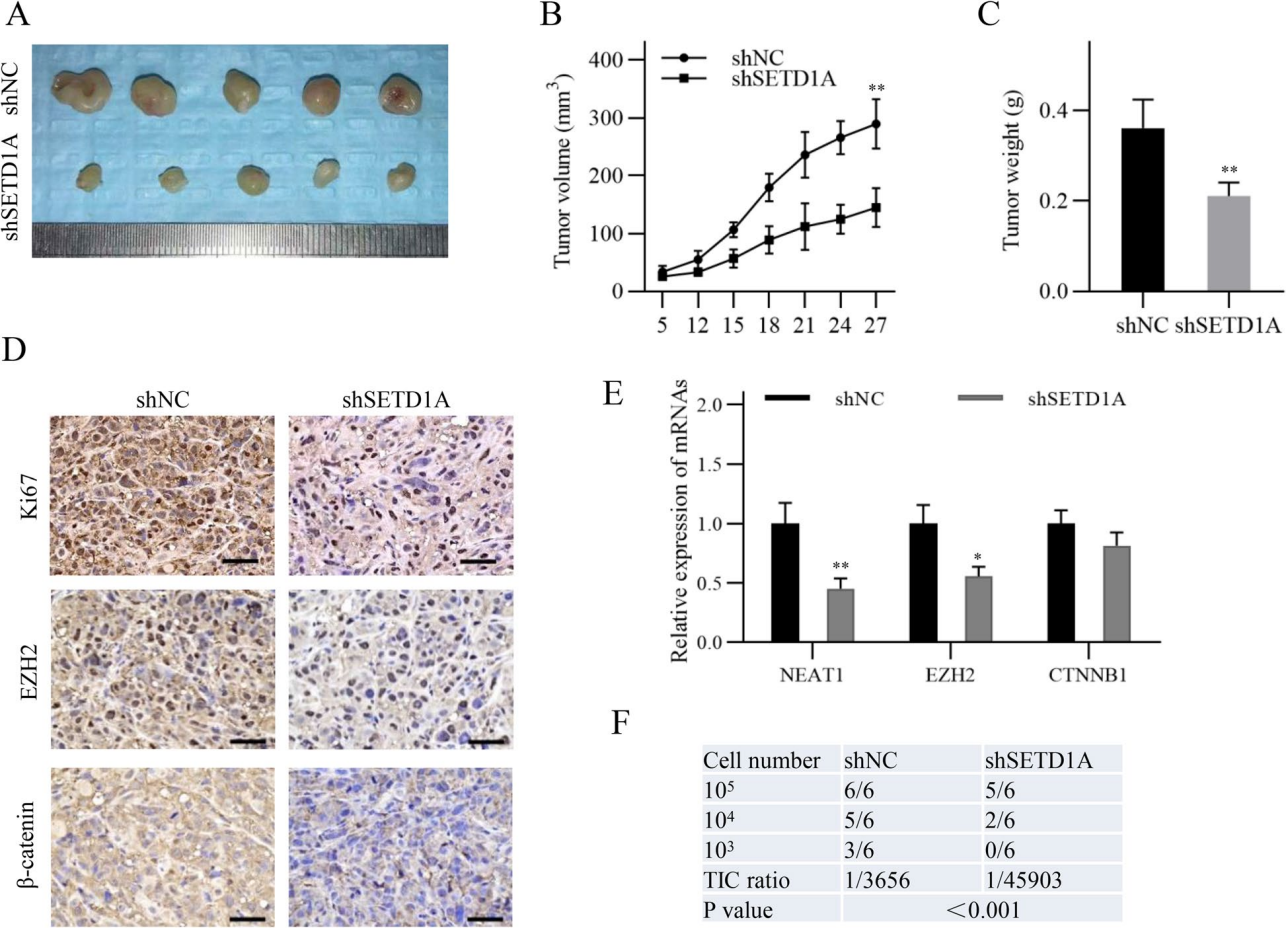
The SETD1A/β-catenin axis promotes NSCLC progression in vivo. **A**, A photograph of the tumors collected from nude mice after 28 days (*n* = 5). **B**, The tumor volumes were measured at the indicated time points. **C**, The tumor weights were measured after xenograft resection. **D**, Immunohistochemical staining of Ki67, EZH2 and β-catenin in the xenograft specimens. Scale bar, 50 μm. **E**, The transcript levels of NEAT1, EZH2 and CTNNB1 in the xenograft tissues were detected by qRT-PCR. **F**, Tumor initiation was monitored for 8 weeks and the tumor initiation frequency was calculated using the ELDA. Data are shown as means ± SD. **P < 0.01.

We performed a nude mouse xenograft assay to validate the role of the SETD1A/NETA1/EZH2/β-catenin axis in NSCLC in vivo. The results showed that the volume and weight of xenograft tumors in the SETD1A-knockdown group were significantly lower than those in the control group (Fig. 6A-C). Consistently, immunohistochemical staining of Ki67 in the SETD1A-knockdown group was significantly reduced compared with that in the control group, indicating a decreased cell growth rate induced by SETD1A knockdown (Fig. 6D). Furthermore, immunohistochemical staining and qRT-PCR assays showed that EZH2, β-catenin and NEAT1 expression were decreased in the SETD1A-knockdown group compared with that in the control group (Fig. 6D-E). In addition, tumor initiating assay showed that SETD1A knockdown reduced tumor initiating cell proportion in NSCLC cells, indicating a critical role of SETD1A in maintaining cancer stem cell property in vivo (Fig. 6F). These results suggest that the SETD1A/NETA1/EZH2/β-catenin axis plays an important role in NSCLC progression.

### SETD1A is a direct target of the Wnt/β-catenin pathway

**Fig. 7:**
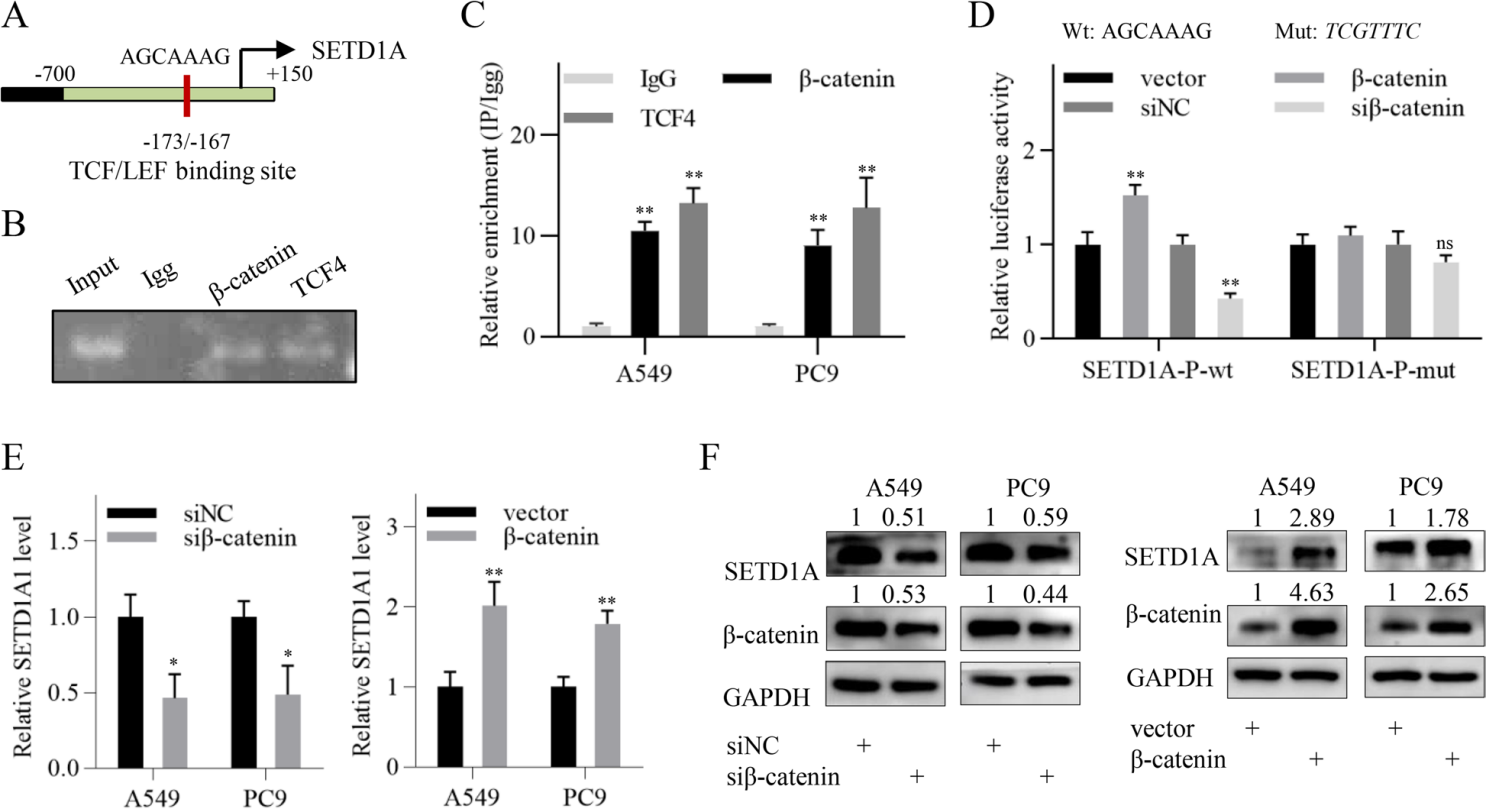
SETD1A is a direct target of the Wnt/β-catenin pathway. **A**, Schematic showing the putative TCF/LEF binding site in the TCF7L2/TCF4 enriched region. Red line, putative TCF/LEF binding site predicted by PROMO online tool; green region, TCF7L2/TCF4 enriched region derived from ENCODE database. **B**, The enrichment of β-catenin and TCF4 in the SETD1A promoter region was detected by ChIP-PCR assay. **C**, The enrichment of β-catenin and TCF4 in the SETD1A promoter region was detected by ChIP-qPCR assay. **D**, The promoter activity of SETD1A gene in NSCLC cells was analyzed by luciferase activity assay. ns, not significant. **E**, SETD1A transcript levels in NSCLC cell as indicated were detected by qRT-PCR. H, SETD1A protein levels in NSCLC cells as indicated were detected by western blotting. Data are shown as the means ± SD. *P < 0.05, **P < 0.01.

**Fig. 8:**
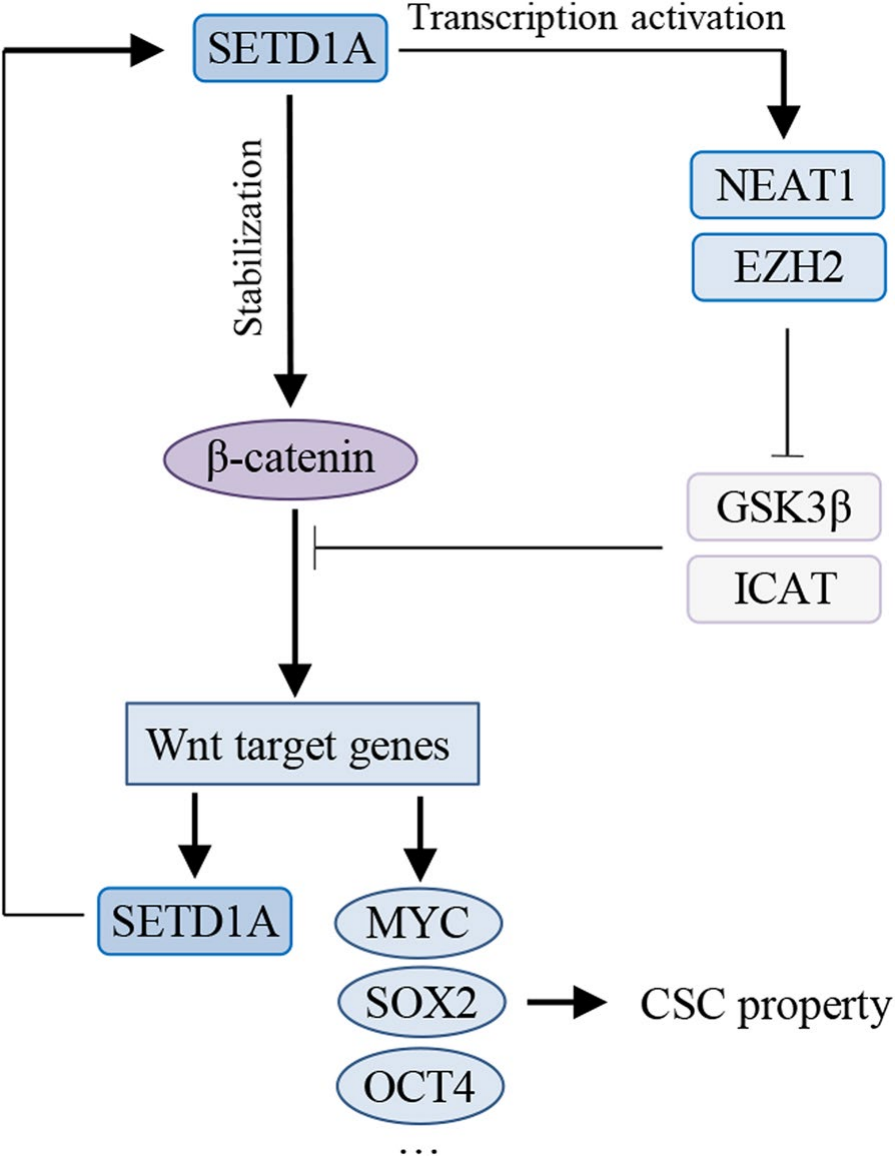
Schematic diagram of the SETD1A/Wnt/β-catenin positive feedback loop in this study. In NSCLC cells, SETD1A activates the Wnt/β-catenin pathway through two mechanisms as follows: i, SETD1A interacts with and stabilizes β-catenin protein; ii, SETD1A promotes the transcription of NEAT1 and EZH2. In turn, the Wnt/β-catenin pathway activates SETD1A transcription, thus forming a positive feedback loop to coordinately promote NSCLC progression

Few studies have elucidated the upstream regulation of SETD1A. Thus, we searched for the evidence of canonical signaling pathways in regulating SETD1A expression in ENCODE database. We found that TCF7L2/TCF4, a β-catenin coregulator, was significantly enriched in the promoter of SETD1A gene according to ChIP sequencing analysis in HeLa-S3, PANC1 and HCT-116 cell lines in ENCODE database, indicating that the Wnt/β-catenin pathway might activate SETD1A transcription (Fig. 7A and Additional file 12: Fig. S10A). By using PROMO database (http://alggen.lsi.upc.es/cgi-bin/promo_v3/promo/promoinit. cgi?dirDB=TF_8.3), we identified a putative TCF/LEF binding site (− 173 to − 167) within the TCF4-enriched region (Fig. 7A and Additional file 12: Fig. S10A). Subsequently, we conducted ChIP with β-catenin and TCF4 antibodies in NSCLC cells. ChIP-PCR showed that both β-catenin and TCF4 were enriched in the SETD1A promoter at the putative TCF/LEF binding sites (Fig. 7B-C). To investigate whether β-catenin affected SETD1A transcription, we constructed two luciferase reporter vectors with the SETD1A promoter fragments (− 360/+ 40) containing the wild-type and mutant putative TCF/LEF binding sites, respectively. The luciferase activity assay showed that β-catenin knockdown significantly inhibited the SETD1A promoter activity, whereas β-catenin overexpression enhanced it (Fig. 7D). However, the luciferase activity of the mutant luciferase reporter vector was not significantly affected (Fig. 7D). As expected, qRT-PCR and western blot analysis showed that β-catenin knockdown reduced the expression of SETD1A transcript and protein, while β-catenin overexpression elevated it (Fig. 7E-F). Mutation of the armadillo repeat domain of β-catenin, including K312E and K435E, abolishes the binding of β-catenin to TCF4 [31–33]. Therefore, we constructed a mutant β-catenin plasmid with K312E and K435E mutations (β-catenin-DM). The results showed that K312E and K435E mutations attenuated the elevated SETD1A expression induced by β-catenin (Additional file 12: Fig. S10B), indicating that the transcriptional regulation of SETD1A by β-catenin occurred in a TCF4-dependent manner. These results suggest that SETD1A is a direct target of the Wnt/β-catenin pathway, forming a positive feedback loop of SETD1A and Wnt/β-catenin to contribute to NSCLC progression (Fig. 8).

## Discussion

Cancer stem cells are associated with cancer recurrence, metastasis and therapy resistance in numerous cancers [34]. In this study, we found that SETD1A plays an important role in regulating cancer stem cell property and cisplatin sensitivity in NSCLC cells. We also found that SETD1A interacts with and stabilizes β-catenin protein to activate the Wnt/β-catenin pathway. Moreover, SETD1A promotes the transcription of NEAT1 and EZH2 in an H3K4me3-depenent manner, leading to an increased Wnt/β-catenin pathway activity. In addition, we demonstrated that SETD1A is a direct target of the Wnt/β-catenin pathway, thus forming a feedback loop of SETD1A/Wnt/β-catenin in NSCLC cells.

It is reported that SETD1A levels are increased in embryonic stem cells (ESCs) and induced pluripotent stem cells (iPSCs), and gradually reduced during the differentiation of stem cells. Further investigation has indicated that SETD1A precisely modulates the pluripotency and self-renewal of stem cells in an H3K4me3-denpent manner [9, 21, 35]. However, another study has reported that SETD1A maintains the self-renewal ability of ESCs in an SET-domain-independent manner [10]. Furthermore, SETD1A deficiency leads to the loss of proliferation capacity and stem cell property in hematopoietic stem cells [36]. In a recent study, SETD1A is proven to promote cancer stem cell property and castration resistance of prostate cancer cells by activating FOXM1 transcription [16]. In this study, we found that SETD1A is frequently overexpressed and predicts a poor prognosis in NSCLC. Furthermore, we demonstrated that SETD1A plays an important role in maintaining cancer stem cell property in NSCLC cells. The ability of cancer stem cells to adopt a quiescent state has emerged as an important driver of drug resistance [34]; thus, we further investigated the effects of SETD1A on chemotherapy sensitivity in NSCLC cells. Our results confirmed that SETD1A reduces the sensitivity of NSCLC cells to cisplatin treatment.

The Wnt/β-catenin pathway is a critical signaling pathway in maintaining cancer stem cell property as previously reported [19]. In the current study, we demonstrated that SETD1A regulated cancer stem cell property and cisplatin sensitivity in NSCLC cells via activating the Wnt/β-catenin pathway. Moreover, SETD1A positively modulated β-catenin protein levels, but the transcript levels were not significantly affected, indicating a potential role of SETD1A in the post-transcriptional regulation of β-catenin. Importantly, SETD1A colocalized with β-catenin not only in the nucleus but also in the cytoplasm. The interaction between endogenous SETD1A and β-catenin in NSCLC cells was further confirmed by co-immunoprecipitation assay. Thus, we speculated that SETD1A might affect β-catenin protein turnover due to its interaction with β-catenin in the cytoplasm of NSCLC cells. Further experiments demonstrated that SETD1A positively regulated β-catenin stability, while negatively regulated ubiquitination of β-catenin. Additionally, S675 phosphorylation induced by PKA might be involved in the mechanism of SETD1A in regulating β-catenin protein ubiquitination and stability in NSCLC cells. However, the role of cytoplasmic SETD1A in cancer progression still needs further investigation.

Many mechanisms are involved in Wnt/β-catenin pathway activity regulation, including many Wnt pathway negative regulators. For example, SIAH1, GSK3β and AXIN2 all take part in the ubiquitination and subsequent proteasome-dependent degradation of β-catenin [19, 37, 38]. DKK1 acts as an LRP6 ligand to inhibit Wnt signaling by preventing Frizzled-LRP6 complex formation [39, 40]. ICAT is involved in β-catenin transcriptional activity by competing for the binding regions of other coactivators, such as TCF4 and P300, resulting in inhibited transcription of Wnt target genes [41, 42]. By analyzing GEO datasets, we noticed that some Wnt pathway negative regulators were upregulated with varying degrees after SETD1A knockdown, including AXIN2, ICAT, SIAH1 and DKK1. Thus, we speculated that SETD1A promotes Wnt/β-catenin pathway activity through inhibiting the expression of these Wnt negative regulators. We identified that ICAT and GSK3β expression in NSCLC cells was increased following SETD1A knockdown. As previously reported, SETD1A generally acts as a positive regulator of its target genes, whether H3K4me3-dependent or -independent [6, 9, 10, 12, 16, 20]. As a result, we speculated that SETD1A probably negatively regulates ICAT and GSK3β indirectly. Based on previous studies and online database, we chose NEAT1 and EZH2 as the putative direct targets of SETD1A for the following two reasons: i, they coordinately activate the Wnt/β-catenin pathway via negatively regulating Wnt pathway negative regulators (AXIN2, GSK3β and ICAT) [30, 43–47]; ii, both the promoters of NEAT1 and EZH2 possess lots of H3K4me3 peaks according to the ChIP sequencing analysis in ENCODE database. Subsequently, we demonstrated that SETD1A activated NEAT1 and EZH2 transcription in an H3K4me3-dependent manner in NSCLC cells. Further experiments suggested that SETD1A promoted Wnt/β-catenin pathway activity and the malignant behaviors in NSCLC cells, at least, partly by upregulating NEAT1 and EZH2 expression.

Many studies have focused on the co-regulators and downstream target genes of SETD1A. However, they have rarely been involved in the upstream regulatory mechanism of SETD1A. We investigated whether canonical signaling pathways regulate SETD1A expression. By using ENCODE database, we identified a few pathways that might be involved in regulating SETD1A expression, including the Wnt/β-catenin pathway. We chose the Wnt/β-catenin pathway for the following experiments due to the possibility of forming a feedback loop with SETD1A. As shown in Fig. 7, we demonstrated that β-catenin and TCF4 bind to the SETD1A promoter and activate SETD1A transcription. Therefore, SETD1A and Wnt/β-catenin pathway formed a feedback loop, which may be a mechanism of maintaining the aberrant SETD1A expression and Wnt/β-catenin pathway activation in NSCLC cells.

## Conclusions

In summary, we found that SETD1A regulates cancer stem cell property and cisplatin sensitivity in NSCLC via activating the Wnt/β-catenin pathway. Moreover, SETD1A interacts with and stabilizes β-catenin to activate the Wnt/β-catenin pathway through the SET domain. Importantly, we identified that NEAT1 and EZH2 are two novel targets of SETD1A, through which SETD1A activates the Wnt/β-catenin pathway activity. In turn, β-catenin activates SETD1A transcription, thus forming a positive feedback loop to promote NSCLC progression. Based on this study, new prognostic factors and therapeutic targets may be determined, contributing to improvements in the diagnosis and prognosis of NSCLC patients.

## Supplementary Information

**Additional file 1: Table S1**. The oligonucleotides used in this study.

**Additional file 2: Table S2**. The clinical and pathological information of patients included in this study.

**Additional file 3: Figure S1**. Normal rabbits IgG staining of NSCLC specimens. Scale bar, upper 100 μm, lower 10 μm.

**Additional file 4: Figure S2.** IC50 value for cisplatin in NSCLC cells as indicated was shown. Data are shown as the means ± SD. *P < 0.05, **P < 0.01

**Additional file 5: Figure S3**. The effects of SETD1A on the Wnt/β-catenin pathway. A, Wnt/β-catenin pathway activity in A549 cells was detected by TOP/FOP flash reporter assay following SETD1A knockdown and overexpression. B, the nuclear β-catenin levels were analyzed by immunofluorescence assay following transfection with the empty vector and SETD1A plasmid. C, CTNNB1 transcript levels in NSCLC cells were detected by qRT-PCR following SETD1A overexpression. D, Cytoplasmic β-catenin levels was detected by western blotting following SETD1A knockdown and overexpression. E, The subcellular localization of SETD1A protein was predicted by the BUSCA webserver. F, The interaction between SETD1A and β-catenin in BEAS2B cells was analyzed by co-immunoprecipitation. G, β-catenin stability was analyzed by CHX chase assay following transfection with the empty vector and SETD1A plasmid. H, Immunofluorescence analysis using normal IgG in PC9 cells is shown. I, β-catenin expression was attenuated by MG132 treatment in SETD1A knockdown cells. J, PC9 cell lysates were immunoprecipitated using a β-catenin antibody and subjected to western blot analysis with the corresponding antibodies as indicated after Wnt3a treatment for 12 h. K, S675 phosphorylation of β-catenin was determined by western blotting following SETD1A overexpression without Wnt3a stimulation. Data are shown as means ± SD. *P < 0.05, **P < 0.01.

**Additional file 6: Figure S4**. SETD1A promotes NSCLC progression via NEAT1/EZH2/β-catenin axis. A, Sphere formation ability in SETD1A knockdown cells was analyzed following transfection with the empty vector, β-catenin, NEAT1 and EZH2 expression vector, respectively. B, Cisplatin sensitivity in SETD1A knockdown cells was analyzed by colony formation following transfection with the empty vector, β-catenin, NEAT1 and EZH2 expression vector, respectively. The final concentration of cisplatin was 5 μM. C, Cisplatin sensitivity was detected by CCK-8 assay following transfection as indicated. Data are shown as means ± SD. *P < 0.05, **P < 0.01. (TIF 192 kb)

**Additional file 7: Figure S5**. SETD1A knockdown increases the expression of Wnt/β-catenin pathway negative regulators. A, AXIN2, ICAT and SIAH1 expression in SETD1A knockdown and negative control group cells in GSE71498 dataset was analyzed. B, DKK1 expression in SETD1A knockdown and negative control group cells in GSE52230 dataset was analyzed. C, AXIN2, ICAT, SIAH1, DKK1 and GSK3β transcript levels in A549 cells were analyzed by qRT-PCR following SETD1A knockdown. Data are shown as means ± SD. *P < 0.05, **P < 0.01. D, DKK1 and AXIN2 protein levels in PC9 cells were analyzed by western blotting following SETD1A knockdown. E, ICAT and GSK3β protein levels in NSCLC cells were analyzed by western blotting following SETD1A knockdown. F, A positive correlation between SETD1A and NEAT1 expression in LUAD tissues was identified in StarBase online database. G, No correlation was identified between SETD1A and NEAT1 expression in StarBase online database. H-I, A positive correlation was identified between SETD1A and EZH2 expression in LUSC (H) and LUAD (I) tissues in StarBase online database.

**Additional file 8: Figure S6**. H3K4me3 peaks in the NEAT1 promoter region in A549 cell line from ENCODE database were visualized in UCSC genome browser.

**Additional file 9: Figure S7**. H3K4me3 peaks in the EZH2 promoter region in A549 cell line from ENCODE database were visualized in UCSC genome browser.

**Additional file 10: Figure S8.** The relative enrichment of WDR5, H3K27ac and H3K27me3 in the NEAT1 and EZH2 promoters was detected by ChIP-qPCR assay. Data are shown as means ± SD. ns, not significant. *P < 0.05.

**Additional file 11: Figure S9**. NEAT1 and EZH2 overexpression attenuates the effects of SETD1A knockdown on the Wnt/β-catenin pathway. A, NEAT1 expression in NSCLC cells transfected with the empty vector and NEAT1 expression vector was analyzed by qRT-PCR. B, EZH2 expression in NSCLC cells transfected with empty vector and EZH2 expression vector was analyzed by western blotting. C, ICAT and GSK3β expression in SETD1A knockdown cells was analyzed by western blotting following transfection with the empty vector, NEAT1 and EZH2 expression vector, respectively. D, Wnt/β-catenin pathway activity in SETD1A knockdown cells was analyzed by TOP/FOP flash reporter assay following transfection with the empty vector, NEAT1 and EZH2 expression vector, respectively. Data are shown as means ± SD. *P < 0.05, **P < 0.01

**Additional file 12: Figure S10**. SETD1A is a target of Wnt signaling pathway. A, ChIP sequencing analysis of TCF7L2/TCF4 in cancer cell lines from the ENCODE database was visualized in UCSC genome browser. B, SETD1A expression was detected by western blot analysis after transfection with the empty vector, wild type β-catenin plasmid and mutant β-catenin plasmid (β-catenin-DM). β-catenin-DM, a mutant β-catenin plasmid with double mutagenesis of K312E and K435E.

## Data Availability

All data generated and analyzed during this study are included in this published article and its additional files.

## Abbreviations

NSCLC: Non-small cell lung cancer
LUAD: Lung adenocarcinoma
LUSC: lung squamous cell cancer
NEAT1: Nuclear Enriched Abundant Transcript 1
DMEM: Dulbecco minimal essential medium
FBS: Fetal bovine serum
siRNA: Small interfering RNA
shRNA: Short hairpin RNA
QRT-PCR: Quantitative Realtime PCR
IHC: Immunohistochemical
ChIP: Chromatin immunoprecipitation
TCGA: The Cancer Genome Atlas
GEO: The Gene Expression Omnibus
CCk-8: Cell Counting Kit-8
