# Supplementary material for: An SETD1A/Wnt/β-catenin feedback loop promotes NSCLC development
Source: J Exp Clin Cancer Res. 2021 Oct 13;40:318. doi: 10.1186/s13046-021-02119-x (PMC8513302; doi:10.1186/s13046-021-02119-x)
Supplement: Supplementary file 1 — Additional file 1: Table S1. The oligonucleotides used in this study. [file 13046_2021_2119_MOESM1_ESM.docx]

Table S1. The oligonucleotides used in this study.

| Name | Sequence (5’-3’) |
| --- | --- |
| qRT-PCR primers | |
| SETD1A-F | TTGCCATGTCAGGTCCAAAAA |
| SETD1A -R | CGTACTTACGGCACATATCCTTC |
| NEAT1-F | CTTCCTCCCTTTAACTTATCCATTCAC |
| NEAT1-R | CTCTTCCTCCACCATTACCAACAATAC |
| EZH2-F | AATCAGAGTACATGCGACTGAGA |
| EZH2-R | GCTGTATCCTTCGCTGTTTCC |
| CTNNB1-F | CATCTACACAGTTTGATGCTGCT |
| CTNNB1-R | GCAGTTTTGTCAGTTCAGGGA |
| ICAT-F | ATGAACCGCGAGGGAGCTCC |
| ICAT-R | CTACTGCCTCCGGTCTTCCG |
| GSK3B-F | GGCAGCATGAAAGTTAGCAGA |
| GSK3B -R | GGCGACCAGTTCTCCTGAATC |
| AXIN2-F | TACACTCCTTATTGGGCGATCA |
| AXIN2-R | TTGGCTACTCGTAAAGTTTTGGT |
| SIAH1-F | TCCAACAATGACTTGGCGAGT |
| SIAH1-R | CTTTTTCTGTGTGTGGCAGAG |
| DKK1-F | CCTTGAACTCGGTTCTCAATTCC |
| DKK1-R | CAATGGTCTGGTACTTATTCCCG |
| ACTB-F | CCTTCTACAATGAGCTGCGT |
| ACTB-R | CCTGGATAGCAACGTACATG |
| ChIP-qPCR primers | |
| NEAT1-P1-ChIP-F | GCCCAGAAACAGCACTACAG |
| NEAT1-P1-ChIP-R | CTTATGAAACCAATGGAGGAGTC |
| NEAT1-P2-ChIP-F | CGCAGGCGAAATGTCTTCAC |
| NEAT1-P2-ChIP-R | AGGACTTTGGACCGTGTAGC |
| NEAT1-P3-ChIP-F | CTGTCCCTCGGCTATGTCAG |
| NEAT1-P3-ChIP-R | CTGCCGGGGTAGAACATTCA |
| NEAT1-P4-ChIP-F | CAACAACATCCGGGAAGAAA |
| NEAT1-P4-ChIP-R | CAGTGTATCCCCGCTTCTCT |
| EHH2-P1-ChIP-F | GACACGTGCTTAGAACTACGAACAG |
| EZH2-P1-ChIP-F | TTTGGCTGGCCGAGCTT |
| SETD1A-P-ChIP-F | TTGACTCCACCAAGGCGGAT |
| SETD1A-P-ChIP-R | TTTGCTTCTCTTCCCCGTCCC |
| shRNA/siRNA target sequences | |
| SETD1A-shRNA1 | GCUGGACGAGUUCUAUAUU |
| SETD1A-shRNA2 | GCUAUGAUGGAGUCCACUU |
| β-catenin-siRNA | AGCUGAUAUUGAUGGACAG |
