# Supplementary material for: An SETD1A/Wnt/β-catenin feedback loop promotes NSCLC development
Source: J Exp Clin Cancer Res. 2021 Oct 13;40:318. doi: 10.1186/s13046-021-02119-x (PMC8513302; doi:10.1186/s13046-021-02119-x)
Supplement: Supplementary file 2 — Additional file 2: Table S2. The clinical and pathological information of patients included in this study. [file 13046_2021_2119_MOESM2_ESM.docx]

Table S2 The clinical and pathological information of the patients in this study

| Number | Age | Sex | Type | Stage | T | N | M |
| --- | --- | --- | --- | --- | --- | --- | --- |
| 1 | 37 | MALE | LUAD | IIIA | T1a | N2 | N/A |
| 2 | 64 | FEMALE | LUAD | IB | T2a | N0 | N/A |
| 3 | 45 | MALE | LUAD | IB | T2a | N0 | N/A |
| 4 | 49 | MALE | LUSC | IV | T3 | N2 | M1b |
| 5 | 64 | MALE | LUSC | IA | T1b | N0 | N/A |
| 6 | 27 | FEMALE | LUAD | IIA | T2a | N1 | N/A |
| 7 | 50 | FEMALE | LUAD | IIA | T2b | N0 | M0 |
| 8 | 70 | MALE | LUSC | IA | T1a | N0 | N/A |
| 9 | 61 | FEMALE | LUAD | IA | T1b | N0 | N/A |
| 10 | 38 | FEMALE | LUAD | IIA | T1b | N1 | N/A |
| 11 | 41 | MALE | LUSC | IIB | T3 | N0 | N/A |
| 12 | 55 | FEMALE | LUAD | IA | T1b | N0 | M0 |
| 13 | 64 | MALE | LUSC | IA | T1b | N0 | M0 |
| 14 | 59 | FEMALE | LUAD | IB | T2a | N0 | N/A |
| 15 | 34 | MALE | LUAD | IA | T1a | N0 | M0 |
| 16 | 47 | MALE | LUAD | IA | T1a | N0 | M0 |
| 17 | 54 | MALE | LUSC | IB | T2 | N0 | N/A |
| 18 | 75 | MALE | LUSC | IIB | T3 | N0 | N/A |
| 19 | 51 | MALE | LUAD | IV | T2b | N0 | M1b |
| 20 | 39 | FEMALE | LUAD | IB | T2a | N0 | N/A |
| 21 | 45 | FEMALE | LUAD | IA | T1a | N0 | N/A |
| 22 | 55 | FEMALE | LUAD | IA | T1b | N0 | M0 |
| 23 | 53 | FEMALE | LUAD | IB | T2a | N0 | N/A |
| 24 | 31 | FEMALE | LUAD | IIA | T2b | N0 | M0 |
| 25 | 36 | MALE | LUSC | IA | T1b | N0 | M0 |
| 26 | 42 | MALE | LUAD | IB | T2a | N0 | N/A |
| 27 | 35 | FEMALE | LUAD | IA | T1b | N0 | M0 |
| 28 | 61 | FEMALE | LUAD | IA | T1b | N0 | N/A |
| 29 | 48 | MALE | LUSC | IB | T2a | N0 | N/A |
| 30 | 54 | MALE | LUSC | IB | T2 | N0 | N/A |
| 31 | 62 | MALE | LUSC | IIIA | T1a | N2 | N/A |
| 32 | 37 | FEMALE | LUSC | IIB | T3 | N0 | N/A |
| 33 | 53 | MALE | LUAD | IA | T1a | N0 | N/A |
| 34 | 50 | FEMALE | LUSC | IIA | T2a | N1 | N/A |
| 35 | 55 | FEMALE | LUAD | IB | T2a | N0 | N/A |
| 36 | 41 | FEMALE | LUAD | IB | T2a | N0 | N/A |
| 37 | 51 | MALE | LUAD | IV | T1a | N1 | M1b |
