# Supplementary figures and images for: An SETD1A/Wnt/β-catenin feedback loop promotes NSCLC development
Source: J Exp Clin Cancer Res. 2021 Oct 13;40:318. doi: 10.1186/s13046-021-02119-x (PMC8513302; doi:10.1186/s13046-021-02119-x)

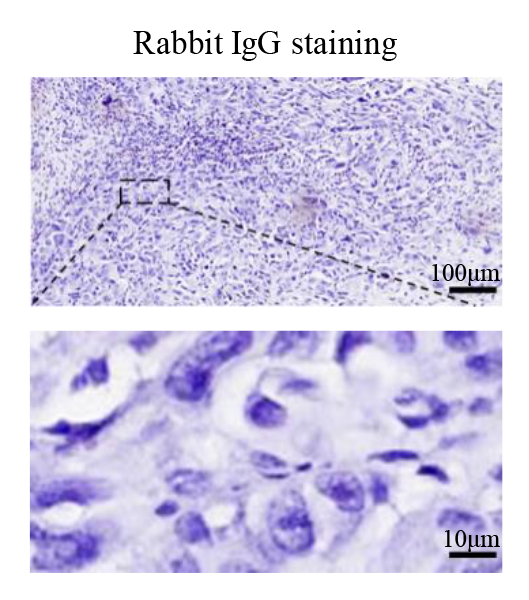

Supplement: Supplementary file 3 — Additional file 3: Figure S1. Normal rabbits IgG staining of NSCLC specimens. Scale bar, upper 100 μm, lower 10 μm. [file 13046_2021_2119_MOESM3_ESM.tif]

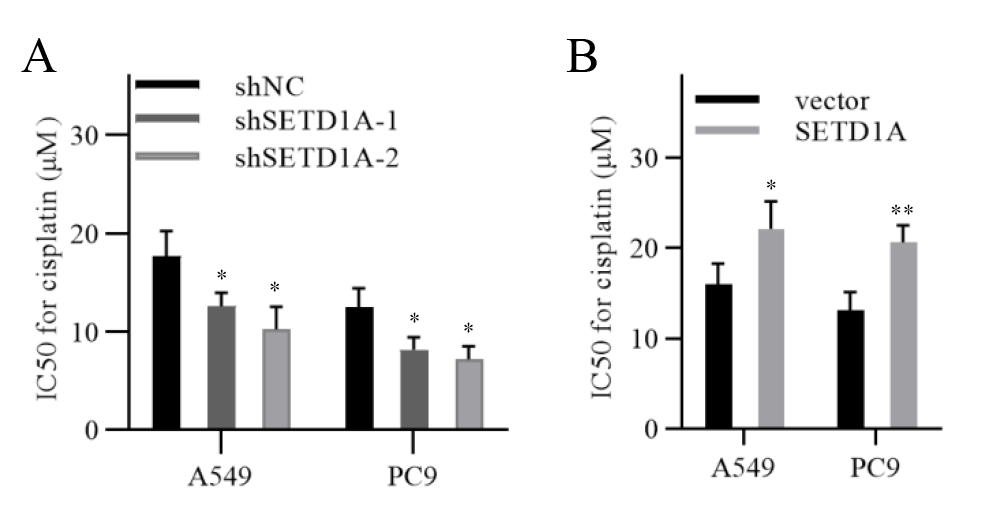

Supplement: Supplementary file 4 — Additional file 4: Figure S2. IC50 value for cisplatin in NSCLC cells as indicated was shown. Data are shown as the means ± SD. *P < 0.05, **P < 0.01 [file 13046_2021_2119_MOESM4_ESM.tif]

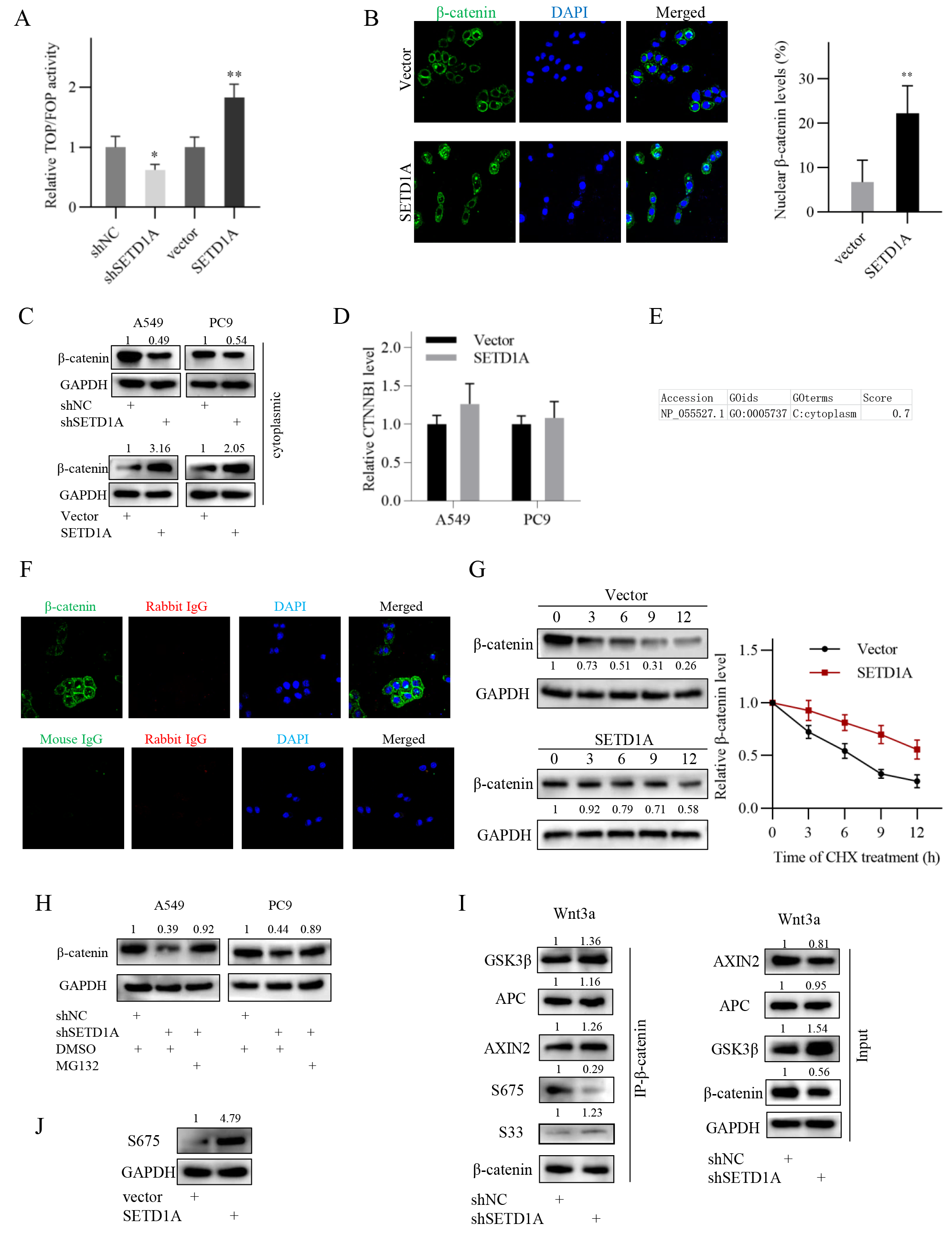

Supplement: Supplementary file 5 — Additional file 5: Figure S3. The effects of SETD1A on the Wnt/β-catenin pathway. A, Wnt/β-catenin pathway activity in A549 cells was detected by TOP/FOP flash reporter assay following SETD1A knockdown and overexpression. B, the nuclear β-catenin levels were analyzed by immunofluorescence assay following transfection with the empty vector and SETD1A plasmid. C, CTNNB1 transcript levels in NSCLC cells were detected by qRT-PCR following SETD1A overexpression. D, Cytoplasmic β-catenin levels was detected by western blotting following SETD1A knockdown and overexpression. E, The subcellular localization of SETD1A protein was predicted by the BUSCA webserver. F, The interaction between SETD1A and β-catenin in BEAS2B cells was analyzed by co-immunoprecipitation. G, β-catenin stability was analyzed by CHX chase assay following transfection with the empty vector and SETD1A plasmid. H, Immunofluorescence analysis using normal IgG in PC9 cells is shown. I, β-catenin expression was attenuated by MG132 treatment in SETD1A knockdown cells. J, PC9 cell lysates were immunoprecipitated using a β-catenin antibody and subjected to western blot analysis with the corresponding antibodies as indicated after Wnt3a treatment for 12 h. K, S675 phosphorylation of β-catenin was determined by western blotting following SETD1A overexpression without Wnt3a stimulation. Data are shown as means ± SD. *P < 0.05, **P < 0.01. [file 13046_2021_2119_MOESM5_ESM.tif]

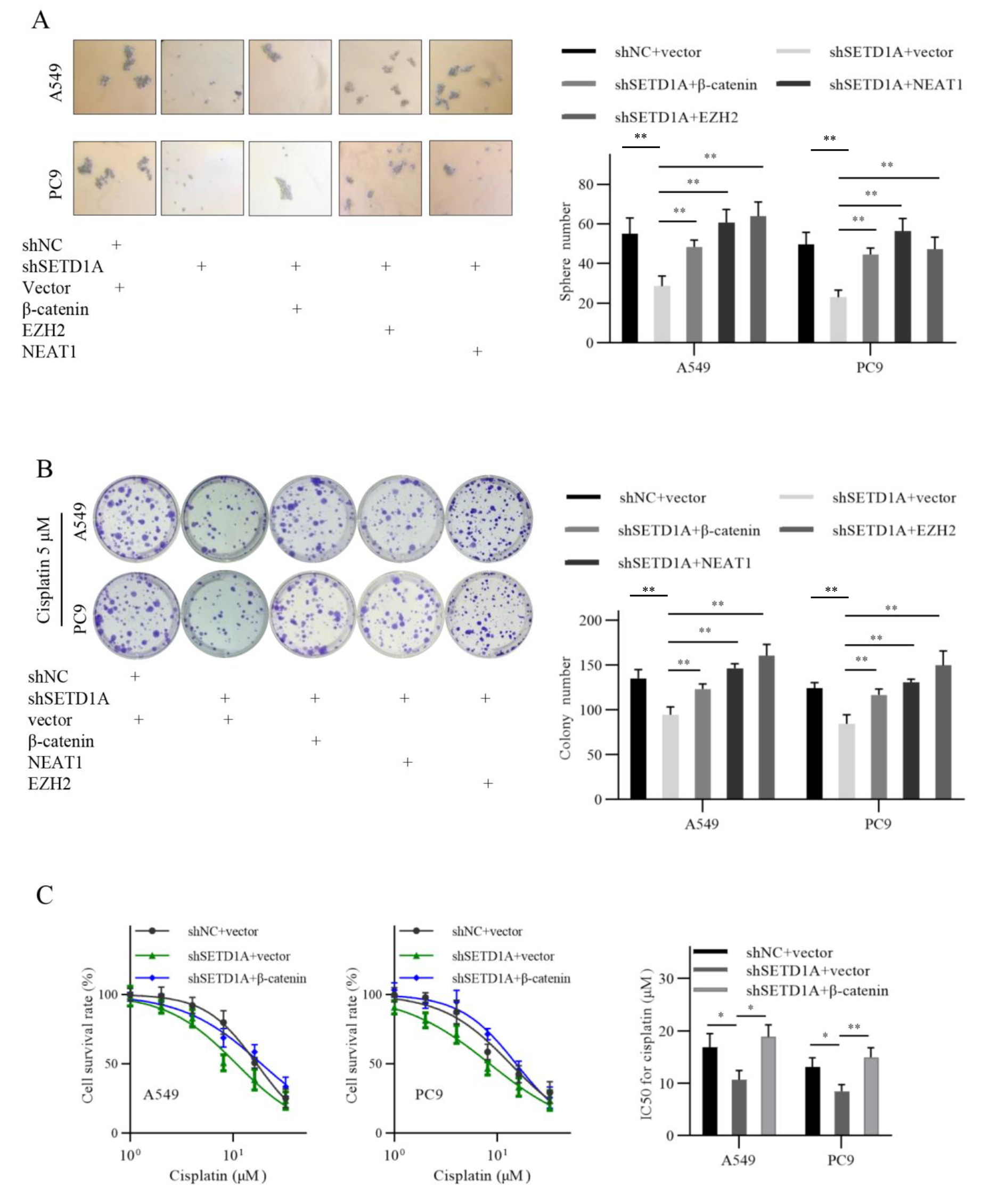

Supplement: Supplementary file 6 — Additional file 6: Figure S4. SETD1A promotes NSCLC progression via NEAT1/EZH2/β-catenin axis. A, Sphere formation ability in SETD1A knockdown cells was analyzed following transfection with the empty vector, β-catenin, NEAT1 and EZH2 expression vector, respectively. B, Cisplatin sensitivity in SETD1A knockdown cells was analyzed by colony formation following transfection with the empty vector, β-catenin, NEAT1 and EZH2 expression vector, respectively. The final concentration of cisplatin was 5 μM. C, Cisplatin sensitivity was detected by CCK-8 assay following transfection as indicated. Data are shown as means ± SD. *P < 0.05, **P < 0.01. (TIF 192 kb) [file 13046_2021_2119_MOESM6_ESM.tif]

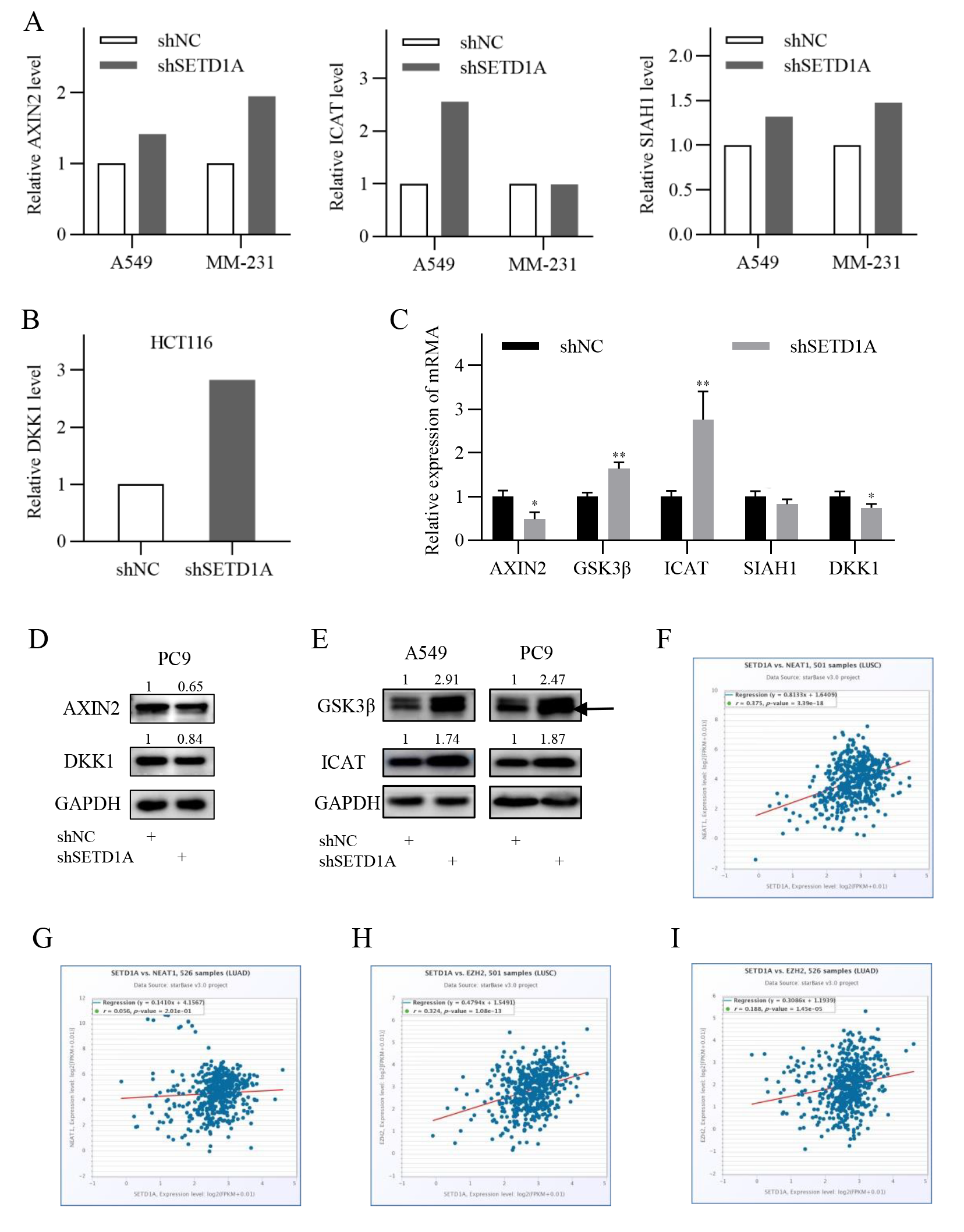

Supplement: Supplementary file 7 — Additional file 7: Figure S5. SETD1A knockdown increases the expression of Wnt/β-catenin pathway negative regulators. A, AXIN2, ICAT and SIAH1 expression in SETD1A knockdown and negative control group cells in GSE71498 dataset was analyzed. B, DKK1 expression in SETD1A knockdown and negative control group cells in GSE52230 dataset was analyzed. C, AXIN2, ICAT, SIAH1, DKK1 and GSK3β transcript levels in A549 cells were analyzed by qRT-PCR following SETD1A knockdown. Data are shown as means ± SD. *P < 0.05, **P < 0.01. D, DKK1 and AXIN2 protein levels in PC9 cells were analyzed by western blotting following SETD1A knockdown. E, ICAT and GSK3β protein levels in NSCLC cells were analyzed by western blotting following SETD1A knockdown. F, A positive correlation between SETD1A and NEAT1 expression in LUAD tissues was identified in StarBase online database. G, No correlation was identified between SETD1A and NEAT1 expression in StarBase online database. H-I, A positive correlation was identified between SETD1A and EZH2 expression in LUSC (H) and LUAD (I) tissues in StarBase online database. [file 13046_2021_2119_MOESM7_ESM.tif]

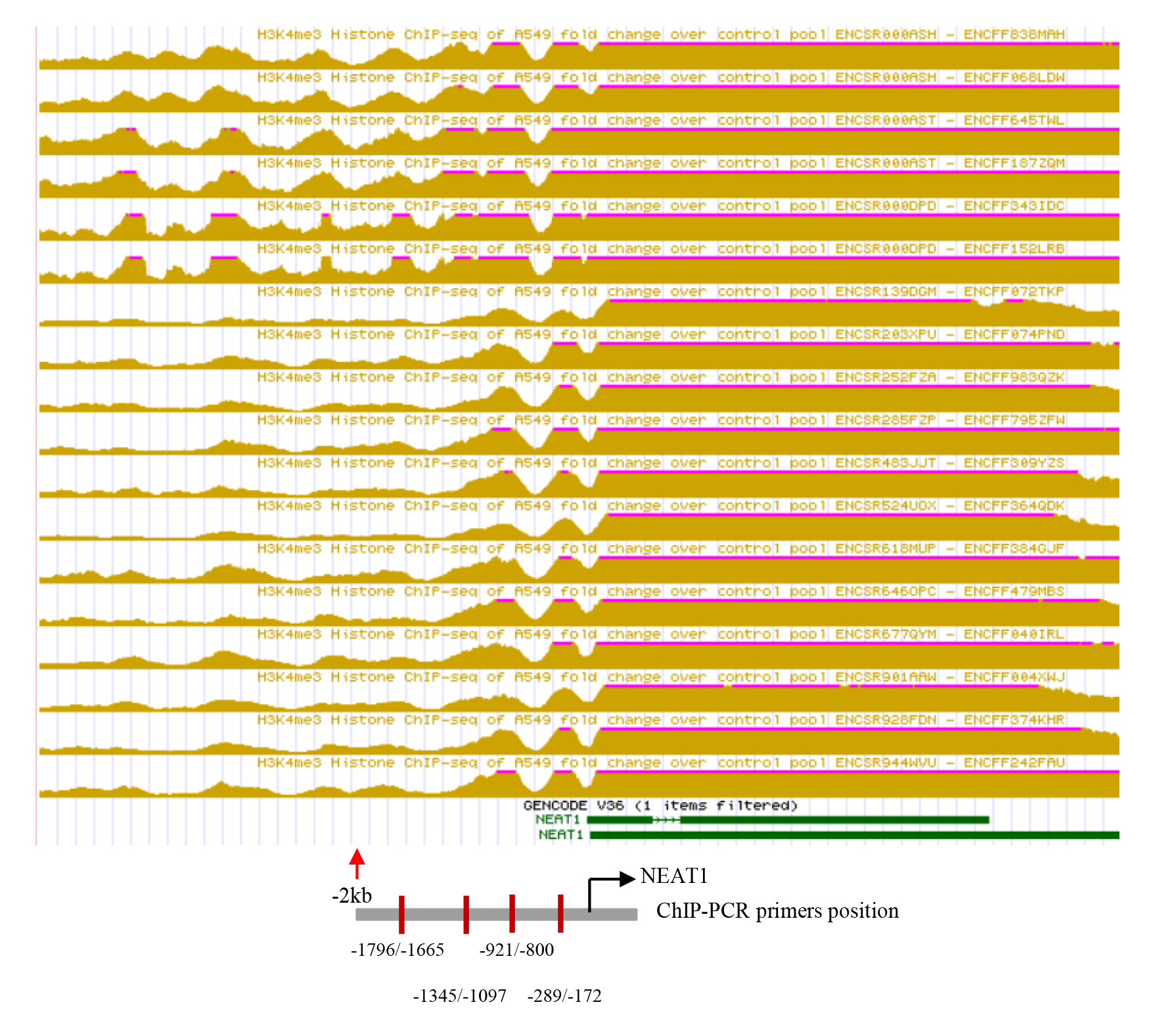

Supplement: Supplementary file 8 — Additional file 8: Figure S6. H3K4me3 peaks in the NEAT1 promoter region in A549 cell line from ENCODE database were visualized in UCSC genome browser. [file 13046_2021_2119_MOESM8_ESM.tif]

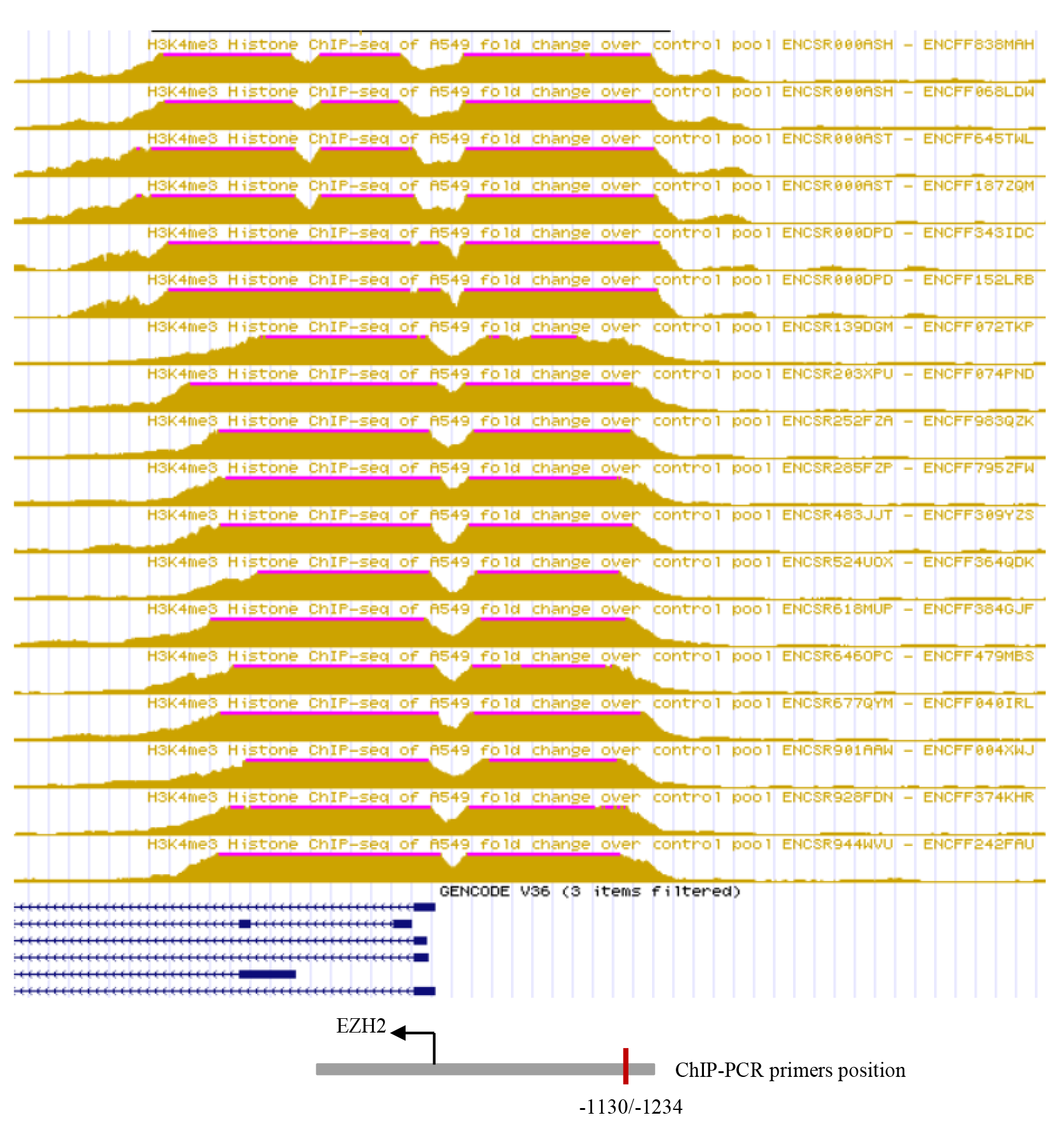

Supplement: Supplementary file 9 — Additional file 9: Figure S7. H3K4me3 peaks in the EZH2 promoter region in A549 cell line from ENCODE database were visualized in UCSC genome browser. [file 13046_2021_2119_MOESM9_ESM.tif]

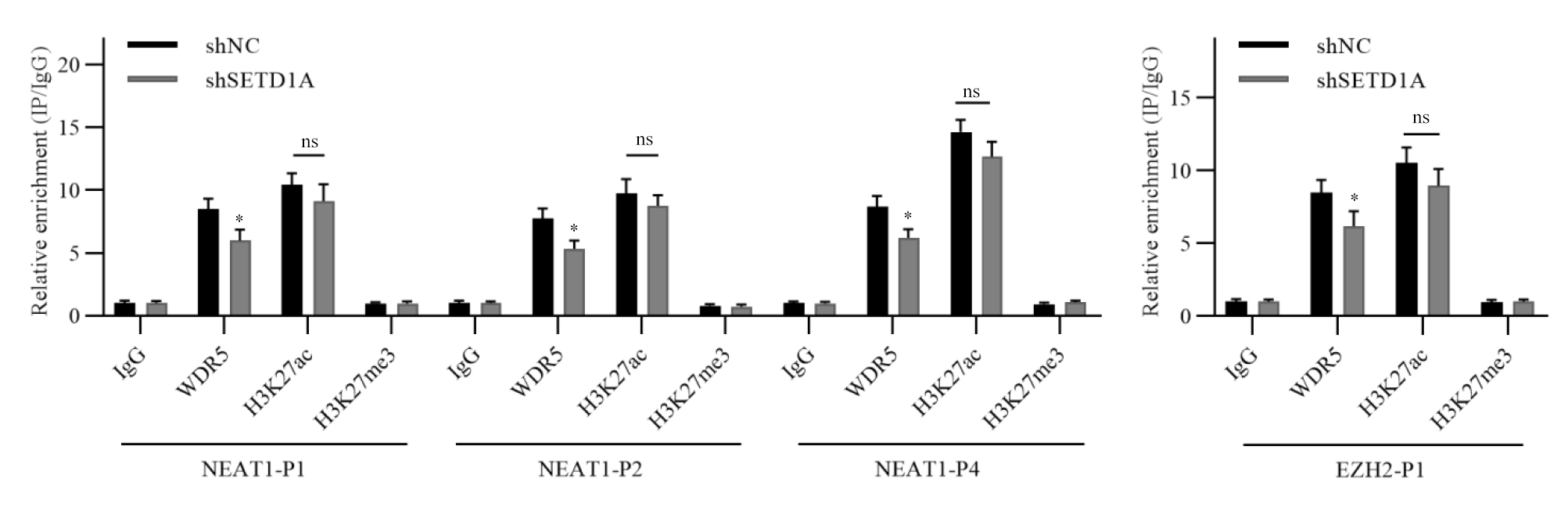

Supplement: Supplementary file 10 — Additional file 10: Figure S8. The relative enrichment of WDR5, H3K27ac and H3K27me3 in the NEAT1 and EZH2 promoters was detected by ChIP-qPCR assay. Data are shown as means ± SD. ns, not significant. *P < 0.05. [file 13046_2021_2119_MOESM10_ESM.tif]

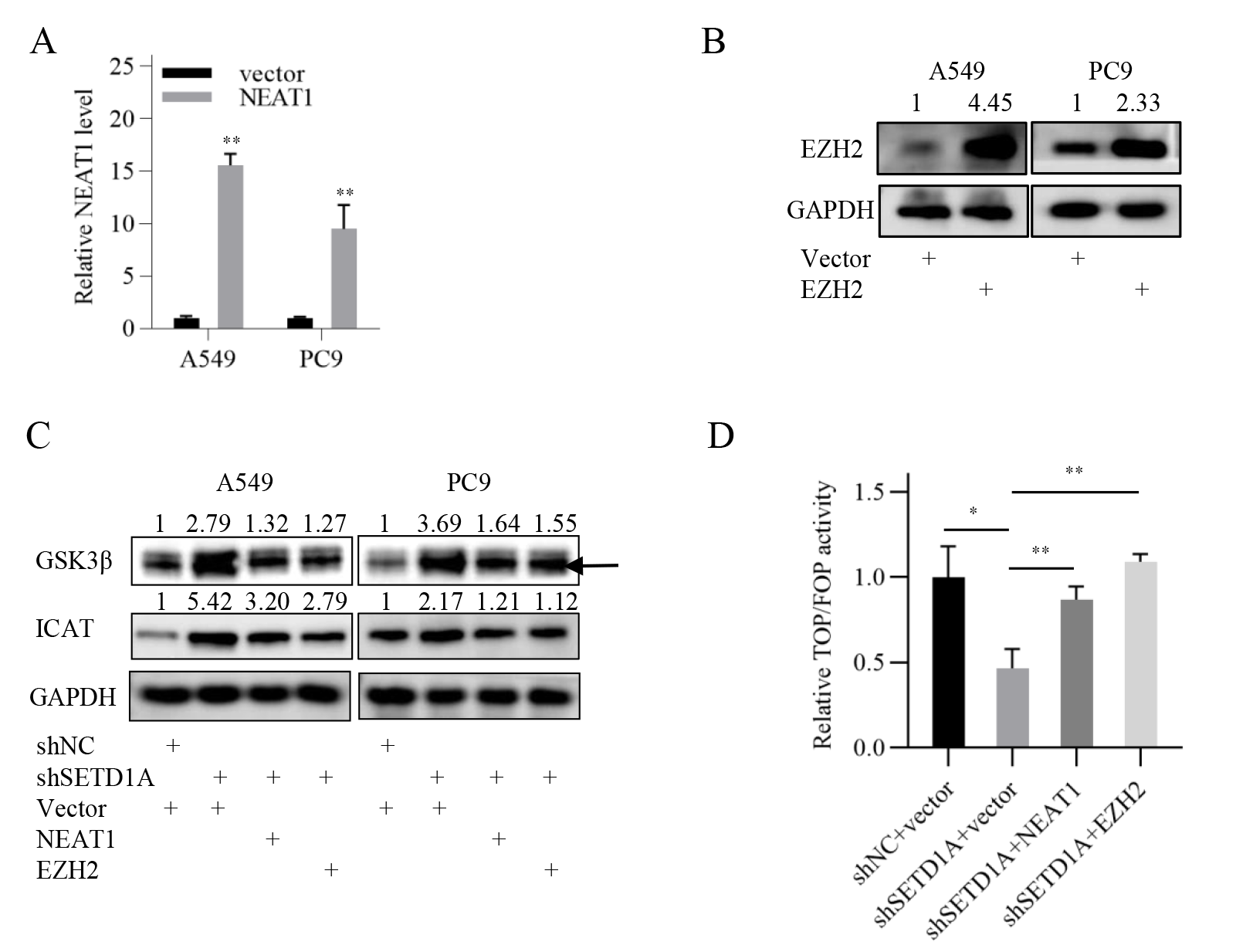

Supplement: Supplementary file 11 — Additional file 11: Figure S9. NEAT1 and EZH2 overexpression attenuates the effects of SETD1A knockdown on the Wnt/β-catenin pathway. A, NEAT1 expression in NSCLC cells transfected with the empty vector and NEAT1 expression vector was analyzed by qRT-PCR. B, EZH2 expression in NSCLC cells transfected with empty vector and EZH2 expression vector was analyzed by western blotting. C, ICAT and GSK3β expression in SETD1A knockdown cells was analyzed by western blotting following transfection with the empty vector, NEAT1 and EZH2 expression vector, respectively. D, Wnt/β-catenin pathway activity in SETD1A knockdown cells was analyzed by TOP/FOP flash reporter assay following transfection with the empty vector, NEAT1 and EZH2 expression vector, respectively. Data are shown as means ± SD. *P < 0.05, **P < 0.01 [file 13046_2021_2119_MOESM11_ESM.tif]

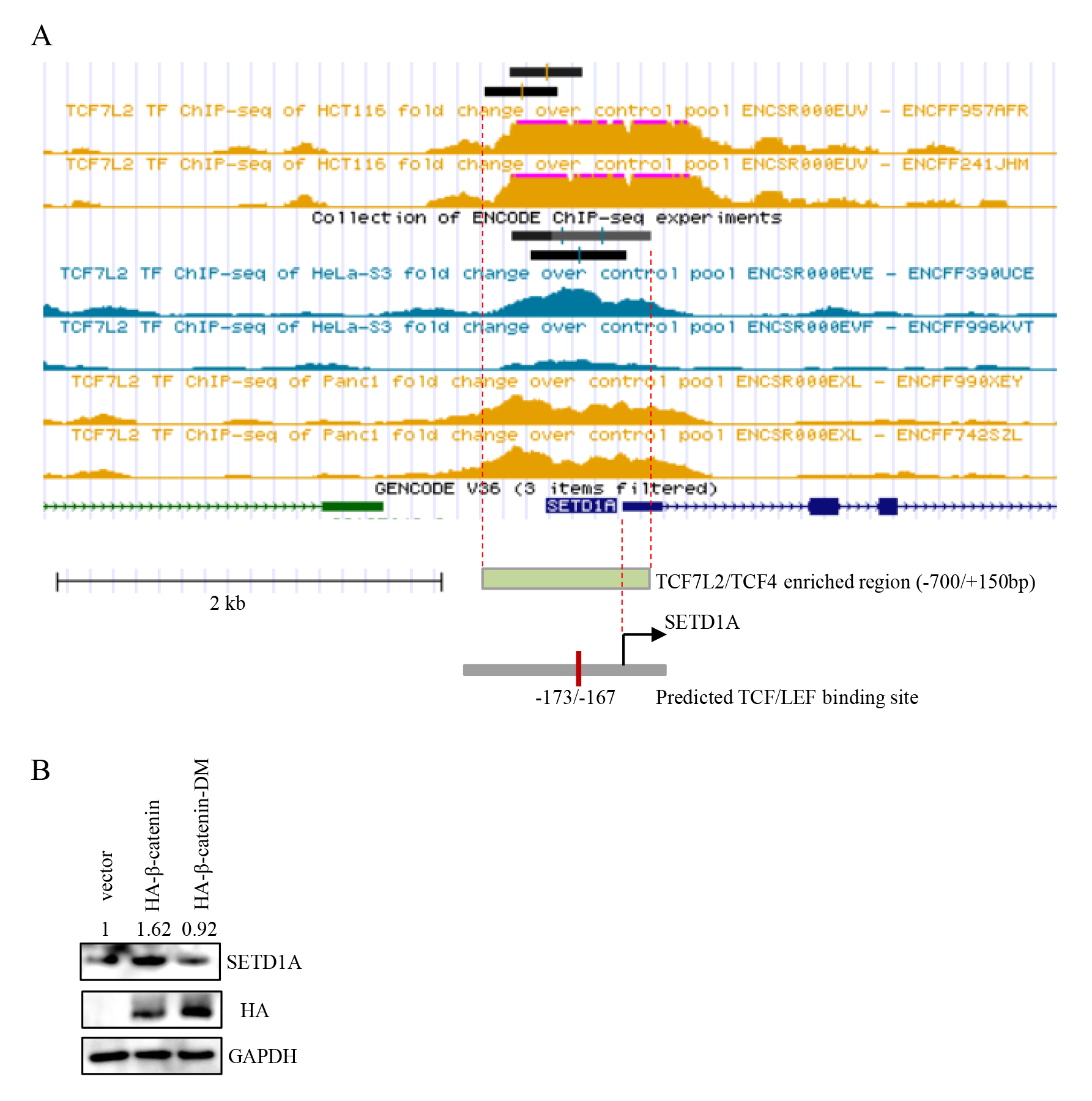

Supplement: Supplementary file 12 — Additional file 12: Figure S10. SETD1A is a target of Wnt signaling pathway. A, ChIP sequencing analysis of TCF7L2/TCF4 in cancer cell lines from the ENCODE database was visualized in UCSC genome browser. B, SETD1A expression was detected by western blot analysis after transfection with the empty vector, wild type β-catenin plasmid and mutant β-catenin plasmid (β-catenin-DM). β-catenin-DM, a mutant β-catenin plasmid with double mutagenesis of K312E and K435E. [file 13046_2021_2119_MOESM12_ESM.tif]
